# DNA-binding mechanism and evolution of replication protein A

**DOI:** 10.1038/s41467-023-38048-w

**Published:** 2023-04-22

**Authors:** Clément Madru, Markel Martínez-Carranza, Sébastien Laurent, Alessandra C. Alberti, Maelenn Chevreuil, Bertrand Raynal, Ahmed Haouz, Rémy A. Le Meur, Marc Delarue, Ghislaine Henneke, Didier Flament, Mart Krupovic, Pierre Legrand, Ludovic Sauguet

**Affiliations:** 1 Architecture and Dynamics of Biological Macromolecules, Institut Pasteur, Université Paris Cité, CNRS, UMR 3528 Paris, France; 2 Univ Brest, Ifremer, CNRS, Biologie et Ecologie des Ecoystèmes marins profonds (BEEP), F-29280 Plouzané, France; 3 Molecular Biophysics Platform, C2RT, Institut Pasteur, Université Paris Cité, CNRS, UMR 3528 Paris, France; 4 Crystallography Platform, C2RT, Institut Pasteur, Université Paris Cité, CNRS, UMR 3528 Paris, France; 5 Biological NMR Platform & HDX, C2RT, Institut Pasteur, Université Paris Cité, CNRS, UMR 3528 Paris, France; 6 Archaeal Virology Unit, Institut Pasteur, Université Paris Cité, CNRS, UMR 6047 Paris, France; 7 Synchrotron SOLEIL, HelioBio group, L’Orme des Merisiers, 91190 Saint-Aubin, France

**Keywords:** Cryoelectron microscopy, Replisome

## Abstract

Replication Protein A (RPA) is a heterotrimeric single stranded DNA-binding protein with essential roles in DNA replication, recombination and repair. Little is known about the structure of RPA in Archaea, the third domain of life. By using an integrative structural, biochemical and biophysical approach, we extensively characterize RPA from *Pyrococcus abyssi* in the presence and absence of DNA. The obtained X-ray and cryo-EM structures reveal that the trimerization core and interactions promoting RPA clustering on ssDNA are shared between archaea and eukaryotes. However, we also identified a helical domain named AROD (**A**cidic **R**pa1 **O**B-binding **D**omain), and showed that, in Archaea, RPA forms an unanticipated tetrameric supercomplex in the absence of DNA. The four RPA molecules clustered within the tetramer could efficiently coat and protect stretches of ssDNA created by the advancing replisome. Finally, our results provide insights into the evolution of this primordial replication factor in eukaryotes.

## Abstract (web summary)

Here the authors present the structure of Replication Protein A (RPA) in Archaea. The RPA structure from P. abyssi has been determined in presence and absence of DNA, providing insights into the evolution of this replication factor in eukaryotes

## Introduction

**Fig. 1:**
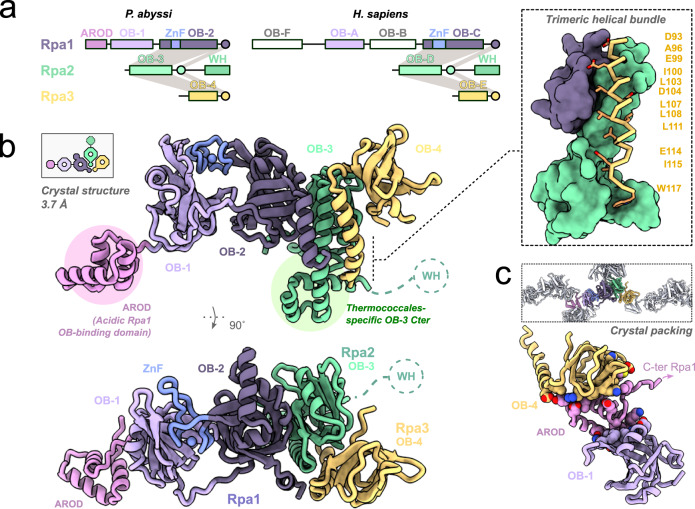
Structure of the archaeal RPA. **a** Domain diagrams of PabRPA and human RPA. **b** Two orthogonal views of the full-length PabRPA crystal structure at 3.7 Å with a focused view on the trimeric helical bundle (top right). **c** Representation of the crystal lattice (top) with a focused view on AROD. Side chains of residues connecting AROD to OB-1 and OB-4 are shown as ball-and-sticks.

In all forms of life, single-stranded DNA-binding proteins (SSBs) are essential components of the DNA replication machinery that primarily protect the exposed single-stranded DNA (ssDNA)^1–3^. They play a vital role in nearly all aspects of DNA metabolism and also act as platforms onto which DNA-processing enzymes can assemble^4,5^. In Bacteria, SSB is the major single-stranded DNA-binding protein^6^. In Eukarya, the ssDNA binding function is primarily achieved by the heterotrimeric Replication Protein A (RPA) complex^7^. In Archaea, the same function is also fulfilled by a heterotrimeric RPA. Composed of three protein subunits, denoted as Rpa1, Rpa2, and Rpa3 (or RPA70, RPA32, and RPA14 in humans), RPA contains multiple OB-folds with different DNA-binding properties (Fig. 1a). Over the past two decades, structural and biochemical studies have mostly been focused on the eukaryotic RPA by using X-ray crystallography^8–13^, NMR^14,15^, SAXS^4^, atomic force microscopy^16^, and cryo-electron microscopy^17^.

While archaeal chromosomes resemble those of most Bacteria, their DNA replication machineries are closely related to their eukaryotic counterparts, serving as powerful models for understanding the function and evolution of the complex eukaryotic replication machineries^18,19^. No structure of archaeal RPA has been determined so far, except for structures of isolated OB domains that have been determined in the framework of structural genomics projects (PDBids: 2K50 and 3DM3). Knowledge of the structure of a full-length archaeal RPA is required to fully understand its cellular function in Archaea. We report three crystal structures and five cryo-EM structures of *Pyrococcus abyssi* RPA (hereafter referred to PabRPA), in its apo form as well as bound to ssDNA. By using an integrative approach that combines X-ray crystallography, cryo-electron microscopy, and extensive biophysical analysis, we investigated the role of each individual domain of PabRPA and uncovered an archaea-specific domain. In the presence of DNA, PabRPA forms filamentous nucleoprotein complexes, which coat and protect ssDNA. In the absence of DNA, PabRPA forms an unanticipated tetrameric supercomplex, which may contribute to RPA clustering in Archaea. This work also clarifies the evolutionary history of RPA, suggesting that the archaeal RPA evolved from a bacterial-like SSB ancestor, and is an evolutionary precursor to the eukaryotic RPA.

## Results

### Architecture of the archaeal RPA heterotrimeric complex

Due to its modular nature, RPA is extremely flexible and can adopt multiple conformations. So far, X-ray crystallography studies have therefore focused on individual domains or truncated RPA trimerization cores (Tri-C)^8–13^. We biochemically reconstituted, crystallized and determined the 3.7 Å X-ray crystal structure of a heterotrimeric complex of *P. abyssi* Rpa1, Rpa2, and Rpa3 subunits (Fig. 1b). Model building was facilitated by determining separately two additional crystal structures of the N-terminal region of Rpa1 (1–180) at 1.8 Å resolution and the Tri-C bound to ssDNA at 3.2 Å resolution (Supplementary Fig. 1 and Supplementary Table 1). Most of Rpa1 (2–358), Rpa2 (1–184), and Rpa3(6-117) subunits have been modeled in the electron density map, the only flexible region being the C-terminus of Rpa2 (185–268). Bioinformatic studies have shown that this region contains a Winged-Helix (WH) domain that is conserved from Archaea to Eukarya^20,21^.

PabRPA contains four OB domains, named OB-1, OB-2, OB-3, and OB-4, which share the highest structural similarity with OB-A, OB-C, OB-D and OB-E in eukaryotic RPA, respectively (Fig. 1a). Like in its eukaryotic OB-C counterparts, PabRPA OB-2 contains a Zn-finger motif that is inserted between β-strands β1 and β2, and a short helical domain that is inserted between β-strands β3 and β4 (Fig. 1b and Supplementary Fig. 2). The Tri-C adopts a compact quaternary structure consisting of OB-2 of Rpa1, OB-3 of Rpa2, and OB-4 of Rpa3, which is the smallest subunit. Similar to the eukaryotic RPA, heterotrimerization of PabRPA is primarily mediated through a three-helix bundle formed by a C-terminal α-helix from each subunit (Supplementary Movie 1). The trimerization helix of Rpa2 is followed by a helix-turn-helix motif, which stabilizes the Tri-C by extending the contacts between the Rpa2 and Rpa3 subunits (Fig. 1b, Supplementary Fig. 2). The N-terminal region of Rpa1 hosts two independent domains, which adopt an extended conformation in contrast to the compact structure of the Tri-C (Fig. 1b, c): the OB-1 DNA-binding domain and an archaea-specific helical domain named AROD (**A**cidic **R**pa1 **O**B-binding **D**omain).

### Structural basis for the high-affinity DNA-binding properties of the PabRPA Tri-C

**Fig. 2:**
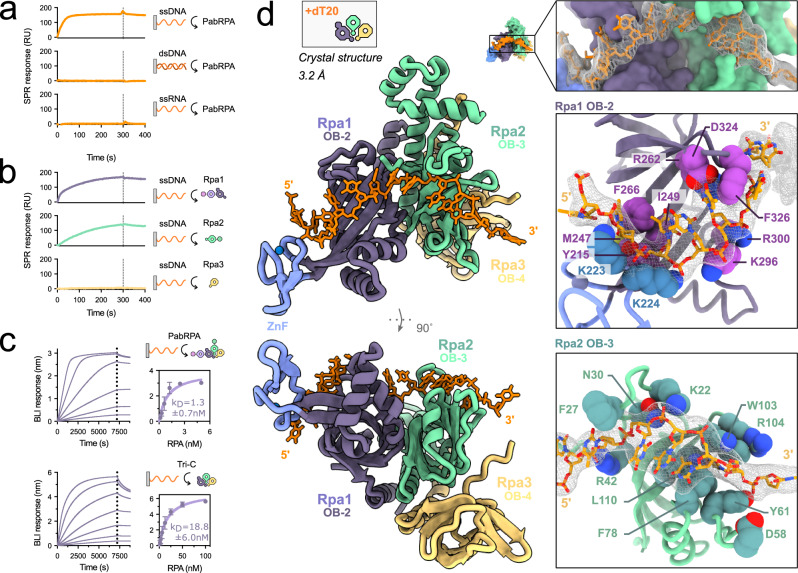
Structural basis for the DNA-binding activity by PabRPA. **a** Nucleic acid-binding specificity for PabRPA. Binding of PabRPA at 5 nM protein concentration on immobilized ssDNA-32mer, dsDNA-32mer, and ssRNA-32mer measured by surface plasmon resonance (SPR) (RU: resonance units). **b** Role of PabRPA subunits on *ss*DNA binding. Specific binding of Rpa1, Rpa2, or Rpa3 at 500 nM on immobilized ssDNA-32mer measured by SPR. **c** Specific binding of PabRPA full-length (5, 2.5, 1.25, 0.63, 0.31, 0.16 nM PabRPA; *n* = 2) or Tri-C (100, 50, 25, 12.5, 6.25, 3.2, 1.6 nM Tri-C; *n* = 3) to immobilized poly-dT35 ssDNA measured by biolayer interferometry (BLI). Steady-state analyses were performed using the average signal measured at the end of the association steps. Data are represented as mean value ± standard deviations (error bars). Source data are provided as a source data file. **d** 3.2 Å crystal structure of the poly-dT20-bound Tri-C, with two focused views on Rpa1 OB-2 and Rpa2 OB-3 ssDNA-interacting residues.

The DNA-binding properties of PabRPA were investigated by electrophoretic mobility shift assays (EMSA), surface plasmon resonance (SPR), and biolayer interferometry (BLI) (Fig. 2a–c, Supplementary Fig. 3). As expected, we found that PabRPA specifically binds to a 24mer or a 32mer random ssDNA, but hardly binds to dsDNA or ssRNA (Fig. 2a). As the use of homopolymeric ssDNA substrates is more convenient for structural studies, we verified that PabRPA binds to a poly-dT ssDNA, with a similar affinity to random ssDNA (1.25 ± 0.6 nM vs 0.71 ± 0.01 nM) (Fig. 2c). Our data show that binding to ssDNA occurs primarily through the Tri-C. Indeed, while a complete PabRPA complex is required for the optimal binding to ssDNA, the Tri-C binds ssDNA with a dissociation constant in the nanomolar range (Kd=18.8 ± 6.0 nM) (Fig. 2c).

To gain insights into the structural basis for the high-affinity interaction between PabRPA and ssDNA, we determined the 3.2 Å crystal structure of the Tri-C bound to poly-dT20 ssDNA (Fig. 2d). The modeled ssDNA lies in a channel that extends from the Rpa1 to the Rpa2 subunits showing 14 contiguous nucleotides that traverse the OB-2 and OB-3 DNA-binding grooves. Rpa3 does not make any contact with the ssDNA in the structure of PabRPA. The DNA-binding properties of each subunit of PabRPA were tested individually by using SPR. While both Rpa1 and Rpa2 subunits individually are capable of binding ssDNA, no interaction was observed for the Rpa3 subunit (Fig. 2b). This property is shared with the eukaryotic RPA where Rpa3 plays a structural role at the heart of the Tri-C but is not involved in ssDNA binding^22–24^.

Comparing the structures of ssDNA-bound PabRPA and the eukaryotic RPA from a fungus *Ustilago maydis*^10^ also reveals intriguing differences (Supplementary Fig. 4). Indeed, although the chemical nature of the interactions and the overall ssDNA binding path through RPA are conserved, the DNA-binding grooves of the respective OB domains are substantially different in Archaea and Eukarya. In the PabRPA structure, basic residues of the β3-β4 helical insertion in the OB-2 make contact with the DNA phosphodiester backbone and delineate a crevice that is narrower than in the eukaryotic RPA (Fig. 2d and Supplementary Fig. 4a). The helical insertion also exists in eukaryotes but adopts a different conformation and is devoid of conserved basic residues, suggesting that it may be an evolutionary relic derived from an archaeal ancestor. On the other hand, the contacts between the OB-2 Zn-finger domain and ssDNA are remarkably conserved between Archaea and Eukarya. Indeed, in the vicinity of their Zn-finger domains, the interacting residues and the two neighboring bases superimpose perfectly.

### A conserved interaction mode in eukaryotic and archaeal RPA-ssDNA complexes

**Fig. 3:**
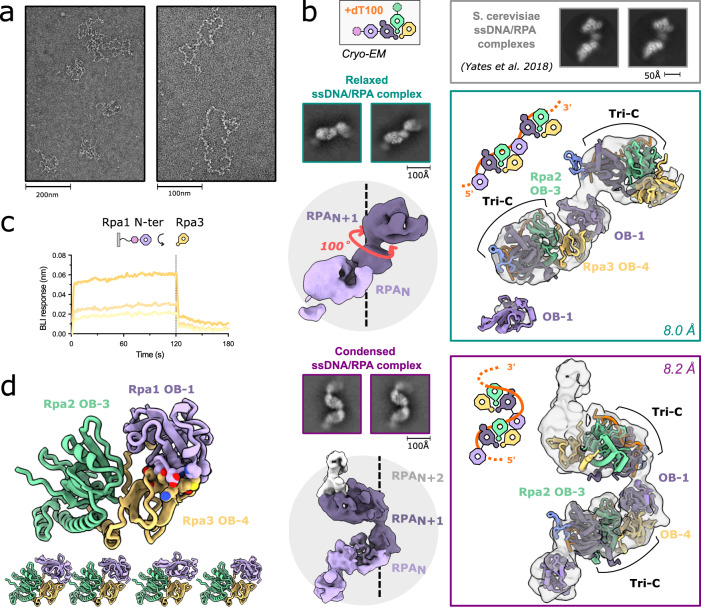
Structural basis for the assembly of multiple PabRPA molecules on ssDNA. **a** Negative-staining microscopy of PabRPA bound to M13 ssDNA plasmid. The experiment was repeated three times independently. **b** Cryo-EM structures of PabRPA bound to a poly-dT100 reveals two interacting modes. Two independent datasets were collected and revealed different levels of condensation of ssDNA-PabRPA complexes: a “relaxed” one and a “condensed” one. Crystal structures of the poly-dT20-bound Tri-C and the OB-1 domain were rigid-body fitted in each cryo-EM 3D reconstruction. Cryo-EM maps are contoured at a level of 6 rmsd. **c** Specific binding of Rpa3 (50, 100, 200 µM; *n* = 3) to immobilized Rpa1-Nter domains (AROD-OB-1) measured by BLI. Source data are provided as a source data file. **d** AlphaFold2 predictions of the Tri-C(*n*)/OB-1(*n* + 1) complex.

We initially assessed the structural basis for the assembly of multiple PabRPA protomers on a long ssDNA substrate by using negative-staining EM (Fig. 3a). PabRPA formed nucleoprotein filaments that coated a 6.4 kb pM13 single-stranded plasmid at a concentration of one PabRPA to ~30 nucleotides. We also collected two independent cryo-EM SPA (single-particle analysis) datasets showing multiple PabRPAs coating the ssDNA by using a poly-dT_100_ oligonucleotide (Fig. 3b, Supplementary Table 2 and Supplementary Fig. 5). Interestingly, 2D class averages reveal that PabRPA dimers adopt two different conformations in the two datasets. In the first dataset, PabRPA protomers adopt a relaxed linear configuration that resembles the one observed for yeast RPA (SceRPA), a study that was conducted using the same poly-dT100 substrate^17^. In the second dataset, PabRPA-ssDNA complexes are more condensed, adopting a pseudo-helicoidal configuration. While the samples were prepared under the same conditions, differences observed in the two datasets are best explained by thinner ice in the first dataset compared to the second.

Both 3D reconstructions show distinguishable structural features (Fig. 3b), such as the trimeric helical bundle, and individual OB domains with the Tri-C. This allowed unambiguous fitting of the DNA-bound PabRPA Tri-C into the EM density map by using rigid-body refinement. 2D class averages show additional density for OB-1 that connects two Tri-Cs from adjacent RPAs. Both 3D reconstructions show that two PabRPA molecules interact with each other on ssDNA through the OB-1 domain of one PabRPA that packs against the Tri-C of an adjacent PabRPA protomer particularly with its OB-4 domain. While their OB-1 domains are similarly oriented in both types of oligomeric forms, the OB-1 domain is more loosely packed to the adjacent Tri-C in the relaxed form than in the condensed one (Supplementary Fig. 6a). Differences between the two DNA-bound PabRPA oligomeric forms are due to a rotation around the OB-1/OB-2 domains of the Rpa1 subunit. In the condensed PabRPA filament, this rotation brings RPA protomers closer together, compared to their position in the relaxed linear filaments observed in PabRPA and SceRPA^17^. Importantly, in both types of filaments, the DNA-binding groove of OB-1 is ideally located to route the ssDNA from an RPA to the other (Supplementary Fig 6b), and protect the DNA from nucleases.

Both extended and condensed DNA-binding modes show that multiple PabRPA molecules are connected together through an interaction between the OB-1 and OB-4 domains of adjacent RPAs, a mechanism that was previously observed for SceRPA^17^.

We performed an AlphaFold prediction of the protein/protein interactions between PabRPA_N_ and PabRPA_N+1_. The prediction showed, with a very high level of confidence, that all five top solutions were superimposable and displayed an interaction between OB-4_N_ and OB-1_N+1_ (Fig. 3d and Supplementary Fig. 7). Importantly, the AlphaFold-predicted protein complex is similar to our 3D reconstructions (Supplementary Fig. 6a). Finally, we tested the interaction of individual Rpa3 subunit with a construct encompassing the Rpa1 N-terminal region (AROD-OB-1) domains using BLI (Fig. 3c). As expected, immobilized Rpa3 interacts with the N-terminal region of Rpa1. Altogether, these results suggest that the multiple RPA molecules are connected together on ssDNA through a conserved interaction in Archaea and Eukarya.

### PabRPA forms a tetrameric supercomplex in the absence of ssDNA

**Fig. 4:**
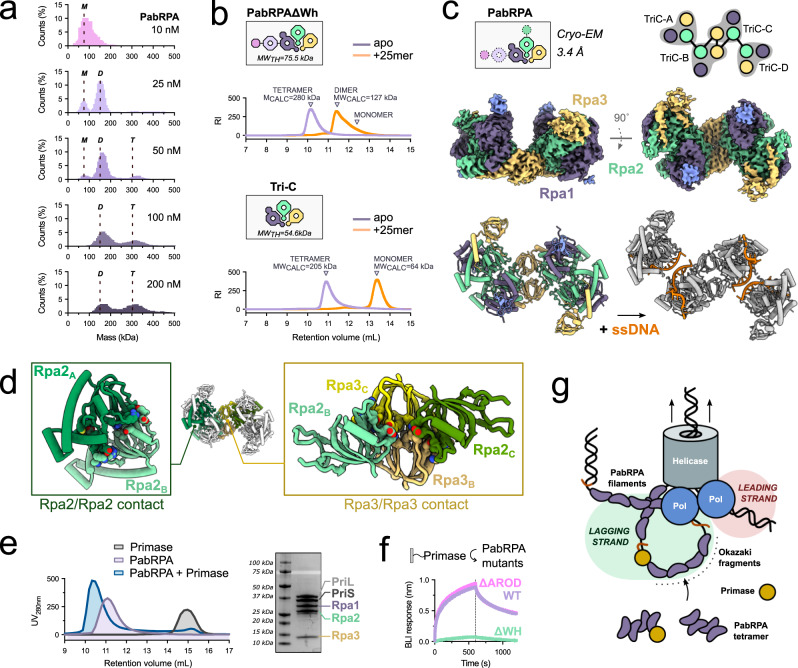
PabRPA assembles as a tetrameric supercomplex that dissociates upon binding to ssDNA. **a** Characterization of PabRPA oligomeric states at low concentrations by using mass photometry. Expected molecular weights for monomeric (M), dimeric (D), and tetrameric (T) PabRPA supercomplexes are indicated with dotted lines. **b** SEC-SLS characterization of PabRPA ΔWH and PabRPA Tri-C in the presence or absence of poly-d25T ssDNA. The theoretical (MW_th_) and calculated molecular weights (MW_calc_) for each complex are given in kDa. PabRPA complexes were injected at 20 µM. **c** 3.4 Å cryo-EM structure of the tetrameric PabRPA super-structure. **d** Focused view on critical contacts within the PabRPA tetrameric assembly. **e** SEC profiles of PabRPA, primase, or PabRPA-primase complexes, injected at 20 µM. The experiment was repeated three times independently. **f** Specific binding of PabRPA, ΔWH, or ΔAROD mutants at 1 µM to immobilized primase measured by BLI. Source data are provided as a source data file. **g** Schematic representation of a DNA replication fork in Archaea. Four PabRPA molecules clustered within the tetramer could efficiently coat and protect the stretches of ssDNA created by the advancing replisome. PabRPA tetramers may also play a role in recruiting and delivering the primase to DNA, thereby contributing to the efficient synthesis of Okazaki fragment, which requires repeated priming by the DNA primase.

Unexpectedly, aside from forming nucleoprotein filaments in the presence of ssDNA, we discovered that PabRPA oligomerizes in solution to form higher-order assemblies in the absence of ssDNA (Fig. 4a). By using mass photometry, PabRPA was shown to form dimers of trimers and tetramers of trimers in a concentration-dependent manner. At micromolar protein concentration range, PabRPA exists predominantly in the tetrameric form, as shown by SEC-SLS (Fig. 4b). SAXS measurement confirmed that PabRPA oligomerizes spontaneously in solution, forming a compact structure with a radius of gyration (Rg) of 57 Å and a Dmax of 207 Å (Supplementary Fig. 8a). To rule out the possibility of buffer-dependent PabRPA oligomer formation, we screened different buffers with a pH ranging from 6 to 9. We observed the same dimeric and tetrameric assemblies of PabRPA, irrespective of the buffer used (Supplementary Fig. 8b). To evaluate the influence of temperature, the light scattering experiment was repeated at 65 °C to get closer to the natural growth temperature of *P. abyssi* (Supplementary Fig. 8c). At this temperature, the tetrameric state of PabRPA was maintained and the intrinsic viscosity increased from 5.0 ml/g at 20 °C to 5.7 ml/g at 65 °C showing no change in the overall compact structure of the tetramer but a small gain in flexibility. In addition, the RPA from *Thermococcus nautili* (62% identical to PabRPA) was expressed, purified, and shown to predominantly exist as dimers and tetramers of heterotrimers in solution, similar to PabRPA (Supplementary Fig. 8d).

To decipher the molecular basis of PabRPA oligomerization in the absence of DNA, the structure of the PabRPA tetrameric supercomplex was determined by cryo-EM at 3.4 Å resolution (Fig. 4c, Supplementary Fig. 9 and Supplementary Table 2). The PabRPA tetrameric supercomplex is formed by two dimers (AB and CD) arranged in a C2-symmetrical assembly, which interact through contacts involving their Tri-Cs. Interactions within the AB and CD dimers involve their OB-3 domains, while the two dimers are held together by interactions involving their OB-4 domains (Fig. 4d). Interestingly, the same OB-3/OB-3 interaction forms a crystal-packing contact in the crystal structure of full-length PabRPA in its apo form (Supplementary Fig. 10). The Rpa1 N-terminal domains are flexible and were not modeled in our 3.4 Å cryo-EM map. This unanticipated oligomerization mechanism is strikingly different from that observed in ssDNA-PabRPA complexes, which involve OB-1 domain.

Notably, many surface residues in the interface that stabilize the PabRPA tetrameric assembly are also found to bind ssDNA. Indeed, most contacts within the AB and CD dimers involve the α1-β1 β-hairpin of OB-3 domain, which also make extensive contacts with ssDNA (Fig. 2b). Consequently, the Tri-C DNA-binding groove, which provides the main contribution to DNA binding in PabRPA, is almost completely occluded in the PabRPA tetrameric supercomplex (Fig. 4c and Supplementary Movie 2). Therefore, dissociation of the PabRPA tetrameric oligomer is required for the Tri-C to bind ssDNA. Consistently, upon addition of a poly-dT25 ssDNA substrate, the PabRPA tetramer is dissociated to DNA-bound dimers and monomers (Fig. 4b). The dimeric form may result from the OB-1/OB-4 interaction between two ssDNA-bound RPA complexes. To test this hypothesis, SEC-SLS experiments were reproduced in the presence or absence of ssDNA by using the PabRPA Tri-C construct, which lacks the OB-1 domain (Fig. 4b). As expected, the PabRPA Tri-C exists as tetramers in solution and upon addition of the poly-dT25 ssDNA substrate, the tetramer dissociates to form monomers but not dimers.

### The PabRPA tetrameric supercomplex interacts with the DNA primase

The RPA tetrameric supercomplexes described above may play important roles in various aspects of DNA metabolism in Archaea. For example, during DNA replication of the lagging strand, a significant amount of ssDNA is present between the progressing helicase and the DNA polymerase. This can pose a problem for high-fidelity DNA replication, as ssDNA is vulnerable to enzymatic and oxidative degradation^25,26^. Four PabRPA molecules clustered within the tetramer could efficiently coat and protect these stretches of ssDNA created by the advancing replisome. Interestingly, Okazaki fragments in *P. abyssi* and other archaeal species have been reported to be of relatively short length (∼100 nucleotides)^27^. Four PabRPA molecules can bind 100-120 nucleotides, thus covering the entirety of the corresponding ssDNA region.

PabRPA tetramers may also serve as molecular platforms to recruit other replication factors. We found that PabRPA tetramers and DNA primase form stable complexes, which can be co-eluted in SEC (Fig. 4e). In addition, we identified the primase-binding site in PabRPA by using BLI (Fig. 4f). DNA primase interacts specifically with the C-terminal region of Rpa2. Bioinformatic studies showed that this region contains a WH domain that also exists in eukaryotes^20,21^. When the region corresponding to the WH domain is deleted, the interaction is lost (Fig. 4f). In order to confirm that DNA primase interacts specifically with the C-terminal region of Rpa2, we performed biolayer interferometry (BLI) experiments using a His_6_-tagged C-terminal region of Rpa2, which was captured via surface-linked Ni-NTA. As expected, the C-terminal region of Rpa2 (Rpa2:180–269) was found to readily bind to the DNA primase, with a *K*_D_ of 126 ± 88 nM (Supplementary Fig. 11).

### Rpa1 hosts an archaea-specific domain involved in protein-protein interactions

**Fig. 5:**
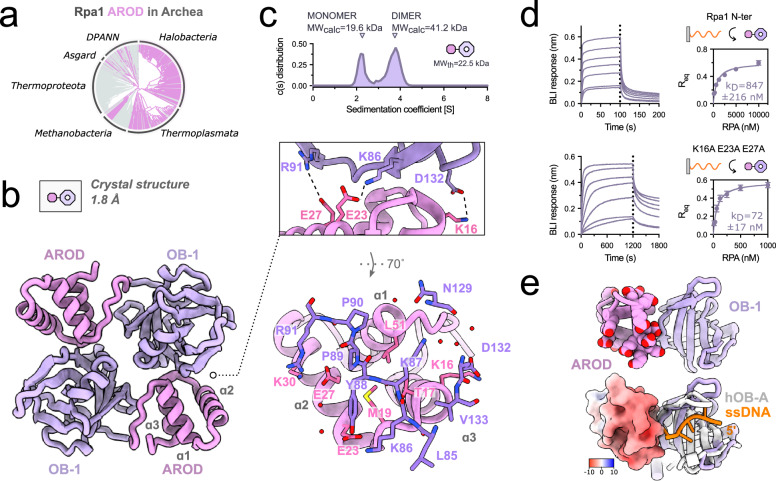
ssDNA binding by OB-1 is modulated by a Rpa1 acidic domain. **a** Phylogenetic analysis (bottom) reveals a broad distribution of Rpa1-AROD domain homologs across Archaea. Notably, in some members of the Thermoplasmata, the AROD domain is found as a standalone domain rather than fused to Rpa1. Genomes lacking AROD domain are presented in gray. **b** High-resolution crystal structure of Rpa1 N-terminal domains at 1.8 Å with a focused view on the AROD/OB-1 dimerization interface. **c** Sedimentation distribution profile of Rpa1-AROD-OB-1 by sedimentation velocity. The theoretical (MWth) and calculated molecular weights (MWcalc) for each complex are given in kDa. **d** Specific binding of WT (10, 5, 2.5, 1.25, 0.63, 0.31, 0.16 µM; *n* = 3) and mutant Rpa1 N-terminal domains (1000, 500, 250, 125, 62.5, 31.3, 15.6 nM; *n* = 3) to immobilized poly-dT35 ssDNA measured by biolayer interferometry (BLI). Steady-state analyses were performed using the average signal measured at the end of the association steps. Data are represented as mean values ± standard deviations (error bars). Source data are provided as a source data file. **e** Focus on the AROD/OB-1 interface. On top, all AROD’s aspartates and glutamates residues are represented as spheres. On bottom, the electrostatic surface potential of AROD is shown with negative, neutral, and positive charges represented in red, white, and blue. The structure of human Rpa1 OB-A bound to ssDNA (1JMC) was superimposed onto the OB-1 domain.

The OB-1 domain of Rpa1 is preceded by an N-terminal tri-helical bundle, which has never been described so far and is referenced in the Pfam database^28^ as a domain of unknown function (DUF2240). This identified domain is absent in eukaryotes, but is broadly present in Archaea, being located at the N-terminal end of Rpa1 and Rpa1-like subunits, with very rare exceptions (Fig. 5a). It is present in nearly all *Thermoplasmatota*, *Halobacteria* and *Asgard*, but absent from *Thermoproteota*, which do not encode Rpa or Rpa-like proteins (Supplementary Fig. 12). Interestingly, in the PabRPA crystal structure, the tri-helical bundle simultaneously stacks against the OB-4 and OB-1 domains of the neighboring Rpa3 and Rpa1 chains, respectively, through a crystal lattice contact (Fig. 1c). We hereinafter refer to this identified domain as the **A**cidic **R**pa1 **O**B-binding **D**omain (AROD).

An interaction between AROD and OB-4 was also observed by Cryo-EM. By isolating a subset of the dataset through 3D classification, we determined an asymmetric 3.8 Å cryo-EM structure of the PabRPA tetrameric supercomplex showing the interaction between the OB-1 domain from one RPA complex and the OB-4 domain of another RPA (Supplementary Fig. 13c). To gain insights into the contacts between AROD and OB-1, the crystal structure of the Rpa1 N-terminal domains AROD-OB-1 was determined at 1.8 Å resolution (Fig. 5b). AROD folds into a three-helix bundle, which is stabilized by a conserved hydrophobic core, and is connected to OB-1 by a 9-aa linker. AROD is an acidic domain, which contains 10 solvent-exposed glutamic acid residues out of 56 residues (Supplementary Fig. 13a). The asymmetric unit of the AROD-OB1 crystal structure shows a C2-symmetric homodimer, which is formed by the interaction between AROD and OB-1 domains. This inter-molecular interaction was already observed in one of the crystal contact of our PabRPA full-length crystal structure (Supplementary Fig. 10). By using analytical ultracentrifugation, we confirmed that the Rpa1 N-terminal region exists in an equilibrium between monomer and dimer in solution (Fig. 5c). The high-resolution view of the AROD-OB-1 interface shows that all three helices of AROD contribute to the interaction with OB-1. The chemical nature of the interactions at the interface is diverse and includes networks of direct and water-mediated buried polar contacts, as well as contacts between hydrophobic residues. Remarkably, the interface includes three salt bridges that involve two acidic residues (E23 and E27) and one basic residue (K17) from AROD (Fig. 5d).

To test the effect of the disruption of this interaction on the DNA-binding properties of OB-1, we compared the binding of wild-type AROD-OB-1 and a triple mutant (K17A-E23A-E27A) to a surface-immobilized poly-dT35 ssDNA by BLI. Strikingly, the AROD-OB-1 triple mutant shows a 10-fold higher affinity for ssDNA than the wild-type construct (*K*_D_ = 72.0 ± 17.0 nM vs 847 ± 216 nM). Furthermore, the wild-type construct showed faster dissociation kinetics than the triple mutant. Indeed, ~60% of the wild-type dissociated from ssDNA in 10 seconds, compared to only 14% for the mutant. Altogether, these results show that interaction with AROD modulates the DNA-binding activity by OB-1. We hypothesize that the acidic AROD domain, which has a high-content of glutamic acid residues, inhibits DNA-binding when it is in contact with OB-1. Consistently, the structure reveals that AROD binds next to the DNA-binding site of OB-1, with its solvent-exposed glutamic acid residues pointing toward the DNA-binding cleft (Fig. 5e).

## Discussion

Coating and protecting exposed ssDNA from nucleases is essential in all forms of life^2^. In Bacteria, the archetypal SSB is the major single-stranded DNA-binding protein^29^. It encompasses a single OB domain and assembles into homotetrameric complexes^30^. Eukaryotes also encode single OB-fold SSBs, but their function is restricted to DNA damage repair, whereas the main ssDNA binding component of the replisome consists of a heterotrimeric RPA complex^4^. In addition to RPA, several RPA-like complexes have evolved to perform various specialized roles, e.g. the CST (Cdc13-Stn1-Ten1) complex, which is essential for telomer maintenance^31–34^.

We find that archaeal and eukaryotic RPAs share a conserved heterotrimerization core, which is composed of three OB domains connected through a three-helix bundle formed by a C-terminal α-helix from each subunit. In addition, the proposed DNA-binding mechanism of PabRPA displays profound similarities with the dynamic model proposed for SceRPA^17^. Archaea and Eukaryotes have evolved a specialized Rpa3 subunit, whose OB domain has lost its DNA-binding abilities^22–24^, but acquired the capacity to interact with the OB-1 domain (OB-A in eukaryotes) of the adjacent RPA molecules in their DNA-bound form. In *P. abyssi*, we observed two forms of ssDNA-RPA assemblies: a relaxed mode with PabRPA molecules arranged in tandem, and a condensed mode with RPA molecules forming a helical-like structure. This dynamic binding of PabRPA, which is highlighted by these two distinct DNA-binding modes, may be necessary in order to achieve RPA’s different cellular functions. This dynamic binding of PabRPA may also be important for enabling its displacement by other replication factors, including polymerases and DNA repair enzymes. Previous biochemical and single-molecule analyses demonstrated that human RPA binds to ssDNA in at least two modes characterized by different dissociation kinetics, including a longer-lived state that is required for DNA repair^35^. The conformations of RPA-ssDNA complexes have been studied using electron microscopy and atomic force microscopy (AFM) imaging, which revealed diverse conformations from amorphous condensates to extended filamentous complexes^16,17,36,37^.

We have demonstrated that RPA from *P. abyssi* and *T. nautili* oligomerize spontaneously in solution to form tetrameric supercomplexes, which may play an important role in various aspects of DNA metabolism in Archaea. Interestingly, a recent study showed that human RPA self-interacts to form dynamic condensates^38^. We hypothesize that the four PabRPA molecules clustered within the tetramer could efficiently coat and protect stretches of ssDNA created by the advancing replisome. Interestingly, Okazaki fragments in *P. abyssi* and other archaeal species have been reported to be of relatively short length (∼100 nucleotides)^27^. Four PabRPA molecules can bind 100-120 nucleotides, thus covering the entirety of the corresponding ssDNA region. This hypothesis is supported by our finding that PabRPA tetramers specifically recruit the DNA primase via a conserved WH domain, and could deliver the primase to the DNA, thereby contributing to the efficient turnover of Okazaki fragments synthesis in Archaea (Fig. 4g). In eukaryotic cells, this WH domain has been shown to recruit a variety of protein factors involved in DNA metabolism^20,21^. Aside from interacting with the DNA primase, the Rpa2 WH domain may also recruit other replication factors in Archaea. Therefore, PabRPA tetramers may serve as molecular platforms to recruit and cluster together a variety of protein factors involved in DNA repair. In eukaryotes, accumulation of RPA signals the presence of damage and activate the DNA damage response^39^. Similarly, accumulation of DNA-free RPA has been shown to inhibit mono-ubiquitination of PCNA by directly interacting with Rad18^40^.

Structures of PabRPA also reveal an α-helical domain which is characterized by a high-content of acidic amino acids: the **A**cidic **R**pa1 **O**B-binding **D**omain (AROD). By itself, AROD is unable to bind either DNA or the primase (Fig. 4f). Instead, AROD participates in RPA-RPA inter-molecular protein-protein interactions. Based on structural and biochemical evidence, including site-directed mutagenesis, we propose that the acidic AROD domain inhibits DNA binding when it contacts OB-1. Importantly, these experiments were performed on isolated domains from the Rpa1 subunit, not on the entire complex. Deleting the entire AROD domain does not significantly increase the affinity of PabRPA for ssDNA (*K*_D_ = 1.0 ± 0.8 nM vs 1.2±0.7 nM) (Supplementary Fig. 13b). This inhibitory mechanism is not an auto-inhibition mechanism but implies that the AROD domain from a DNA-free RPA molecule would partially dissociate a DNA-bound RPA molecule, in the OB-1 region. Indeed, the AROD-OB-1 linker is too short to enable auto-inhibition of OB-1. Interestingly, real-time single-molecule studies on SceRPA, revealed that RPA rapidly dissociates from ssDNA when free RPA is present in solution allowing rapid exchange between the free and bound states. In contrast, SceRPA remains bound to ssDNA for longer periods of time when free protein is absent from the solution^41^. Such an exchange mechanism, involving AROD and DNA-free RPA tetramers may also exist in Archaea, and may play an important role in regulating the association and dissociation of RPA from ssDNA in Archaea. Indeed, a crucial feature of RPA is that, while being able to bind nucleic acids with very high-affinity, it must be readily displaced to hand over ssDNA to other enzymes for further downstream processing^17^. In this sense, interaction between AROD and OB-1 may generate a partially dissociated intermediate, which exposes a small section of ssDNA allowing other proteins to access DNA. FRET-based solution studies on SceRPA revealed dynamic rearrangements of OB domains during coordinated RPA binding, which are regulated by phosphorylation on OB-A, the equivalent of archaeal OB-1 in eukaryotic RPA^17^. A recent study on human RPA showed that RPA self-interactions are regulated by multi-site phosphorylations^38^. Their study suggests that nuclear RPA condensates provide a reservoir of highly concentrated free RPA in excess over the bound ssDNA, which enables rapid exchange of RPA molecules on the enclosed ssDNA. Similarly, AROD may participate in a primitive regulation mechanism of DNA-binding in Archaea.

While AROD is widely distributed among Archaea, it is absent in eukaryotes. Instead of AROD, the eukaryotic Rpa1 N-terminal extension includes an extra OB domain that is also dedicated to protein-protein interactions and is linked to OB-A by a 50 amino-acid long linker with no predicted secondary structures (Supplementary Fig. 13d). However, PabRPA and the eukaryotic CST complex share intriguing similarities. First, the Ctc1 subunit of the Rpa-like CST complex hosts a three-helix domain, termed the ‘hinge’, which resembles AROD^32^. While we could not find any sequence conservation, it is striking that the hinge domain has been shown to segregate several OB domains, similar to AROD. Second, CST associates with the Polα-primase primosome to form pre-initiation complexes, like RPA tetramers that form stable complexes with DNA primase in archaea^42^. Finally, similar to PabRPA which oligomerizes in solution, the structure of the human CST complex revealed a higher-order decameric assembly bound to telomeric DNA^32^.

**Fig. 6:**
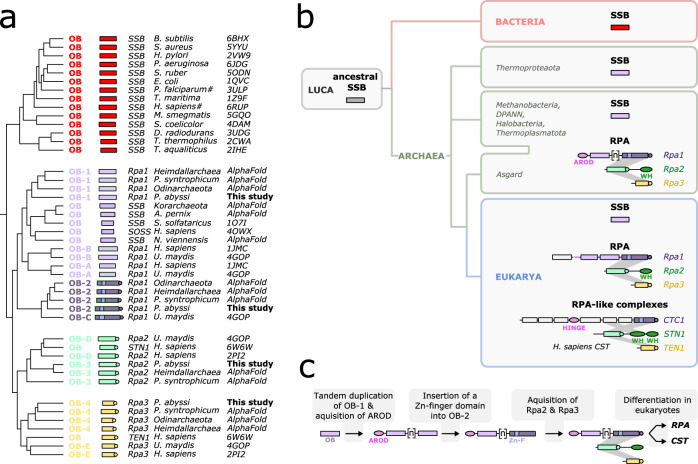
Evolutionary implications for the RPA evolution in Archaea and Eukarya. **a** Structure-based dendrogram produced by DALI, based on average linkage clustering of the Z-scores of the structural similarity matrix. The available structures were supplemented with AlphaFold models^60^ of additional crenarchaeal and thaumarchaeal SSBs and trimeric RPAs from Asgard archaea, the postulated ancestors of eukaryotes^76,77^. **b** Current proposal for the evolutionary history of SSB and RPA in the three domains of life. **c** Putative evolutionary scenario suggesting that the archaeal RPA evolved from a bacterial-like SSB ancestor, and is an evolutionary precursor to the eukaryotic RPA and CST.

Prior to this work, little was known about the structure of RPA in Archaea and most biochemical or genetic studies involved knocking-out entire genes^43^. Knowledge of the domain architecture of the different RPA subunits in Archaea, will serve as a basis for investigations of the biological functions of individual domains or specific mutations. Archaea display a patchier distribution of SSB and RPA proteins than eukaryotes counterparts^3,20,44–46^, with some archaeal lineages encoding either one or both systems^47,48^. Such SSB/RPA distribution patterns prompt questions regarding the emergence of the trimeric RPAs and the origin of the different eukaryotic ssDNA binding proteins. We performed an all-against-all structural comparison of the OB domains from representative SSB and RPA originating from Bacteria, Eukarya, and Archaea as well as from the eukaryotic RPA-like CST complex (Fig. 6a). The structures formed three major clades in the structural distance-based tree. The first clade included all bacterial SSBs. In the second clade, archaeal as well as eukaryotic SSBs are grouped with the OB-1 domain of Rpa1 (OB-A in eukaryotes), while the OB-2 of Rpa1 (OB-C in eukaryotes) forms a sister group to this assemblage. Finally, the third clade included two sister subclades corresponding to the OB-3 and OB-4 domains of archaeal and eukaryotic Rpa2 and Rpa3, respectively. It was previously noted that it is difficult to distinguish single-OB domain SSBs and Rpa3^3^. Structural comparisons clearly distinguish the two sets of proteins and strongly suggest an evolutionary relationship between the archaeal SSBs and the N-terminal OB domain of archaeal and eukaryotic Rpa1 subunits, rather than with Rpa3 (Fig. 6b, c). These observations are consistent with the evolution of heterotrimeric RPA from a single-OB SSB ancestor, likely within the archaeal branch^49–51^. Given the shared non-structured C-terminal extension in bacterial and archaeal SSBs and their ability to oligomerize, it is likely that they have evolved from a common ancestor, which might have been functional already in the last universal cellular ancestor (LUCA). Within archaea, this SSB gave rise to the multidomain Rpa1 through tandem duplication followed by the insertion of a Zn-finger subdomain into OB-2. By contrast, Rpa2 and Rpa3 appear to be structural paralogs, with Rpa2 accruing a characteristic C-terminal WH domain. Interestingly, the PabRPA OB-3 and OB-4 domains respectively share the highest structural similarity with the OB-domains from the Ctc1 and Ten1 subunits of the human telomeric maintenance CST complex. More generally, the OB domains from eukaryotic RPA and RPA-like complexes, such as the CST, are seen to be grouped with the corresponding domains of archaeal PabRPA-like homologs, including those from Asgard archaea, suggesting that the eukaryotic RPA and its derivatives have been inherited from archaeal ancestors. The strong structural similarity observed between PabRPA and the human telomeric maintenance CST complex is also intriguing. Indeed, telomer maintenance in linear genomes is a defining feature of eukaryotes compared to prokaryotes, which possess circular genomes. Therefore, the inherited ancestral RPA may have provided the primitive proto-eukaryotes with key genome maintenance activities that were required for the emergence of the eukaryotic domain of life.

## Methods

### Cloning, protein expression, and purification

The open reading frames (ORFs) of the Rpa1, Rpa2, and Rpa3 genes from *P. abyssi* and *T. nautili* were optimized and synthesized by GeneArt (Thermo Fisher). For the expression of individual subunits, ORFs were inserted into the pRSFduet(+) (Novagen) multiple cloning site 1 with a TEV-cleavable N-terminal 14-His tag. For the co-expression of PabRPA complexes, ORFs were inserted into the pRSFduet(+) multiple cloning site 1 as a polycistronic Rpa3-Rpa2-Rpa1 construct separated by ribosome binding sites (RBS), with a TEV-cleavable N-terminal His14-tagged Rpa3 fusion protein. The folowing RPA isoforms were cloned from the pRSFduet(+) constructs using the Q5 site-directed mutagenesis kit (New England Biolabs): PabRpa1 AROD-OB-1 (Rpa1_1-193_), PabRpa1 AROD-OB-1 K16A E23A E27A triple mutant, PabRPA trimerization core Tri-C (Rpa1_193-358_/Rpa2_1-179_/Rpa3), PabRPA ΔAROD (Rpa1_64-358_/Rpa2/Rpa3), PabRPA ΔAROD-OB-1 (Rpa1_193-358_/Rpa2/Rpa3), PabRPA ΔWH (Rpa1/Rpa2_1-179_/Rpa3), PabRPA WH (Rpa2_180-269_). The ORFs of the PriS and PriL genes from *P. abyssi* were optimized and synthesized commercially by GeneArt (Thermo Fisher) and inserted into pRSFduet(+) with a TEV-cleavable N-terminal His14-tagged PriS fusion protein

Proteins were expressed in the *E. coli* BL21 Star (DE3) strain (Invitrogen) at 37 °C in LB medium supplemented with 100 μg/mL of kanamycin. Recombinant protein expression was induced by adding 0.25 mM IPTG. Cells were then incubated overnight at 20 °C, collected by centrifugation, resuspended in buffer A (0.02 M Na-HEPES (H3375, Sigma) at pH 8, 0.5 M NaCl (S9888, Sigma), 0.02 M imidazole (I2399, Sigma)) supplemented with complete EDTA-free protease inhibitors (A32965, Thermo Scientific), and lysed with a Cell-Disruptor (CellD). Lysates were then heated for 10 min at 60 °C and centrifuged 30 min at 20000 g. PabRPA purifications were performed using a three-step protocol including nickel affinity, anion exchange, and size exclusion chromatography. The clear cell lysate was loaded onto 5 mL HisTrap columns (Cytiva) connected to an ÄKTA purifier (Cytiva). Elutions were performed using a linear gradient of imidazole (buffer B, 0.02 M Na-HEPES at pH 8, 0.5 M NaCl, 0.5 M imidazole). Protein fractions were combined, dialyzed in buffer C (0.02 M Na-HEPES pH 8, 0.1 M NaCl), loaded onto 5 ml HiTrap Q FF columns (Cytiva), and eluted with a linear gradient, by mixing buffer C with buffer D (0.02 M Na-HEPES pH 8, 2 M NaCl). Depending on the applications, the 14-His tag was removed following an overnight TEV-protease cleavage. Purifications were finally polished using exclusion-size chromatography in buffer E (0.02 M Na-Hepes pH 8, 0,15 M NaCl) on a Superdex 200 10/300 (Cytiva) column.

### Crystallization

Crystallization trials were performed using the hanging drop vapor diffusion technique in 2 μL drops (1:1 reservoir to protein ratio) equilibrated against 500 μL of reservoir solution. PabRPA crystals were obtained at 18 °C in 0.1 M Tris pH 8.5, 1.2 M ammonium sulfate (A4418, Sigma) with a protein solution at 10 mg/mL. After 72 hours, crystals were transferred in a dehydration solution containing the original mother liquor supplemented with 25% glycerol (BP220, Fischer) and incubated for 1 hour at 4 °C before flash-freezing in liquid nitrogen. PabRpa1 AROD-OB-1 crystals were obtained at 18 °C using a crystallization solution made of 0.01 M NiCl2, 20% w/v PEG MME 2 K, and 0.1 M Tris pH 8.5 with a protein solution at 40 mg/mL. Crystals were then directly flash-frozen in liquid nitrogen without additional cryoprotection. The PabRPA Tri-C/dT20 complex was reconstituted by mixing the protein solution at 0.5 mg/mL with a 1.2× excess of poly-dT20 ssDNA (Eurogentec) in buffer E. The mixture was then concentrated ~30 times to obtain a final OD_280nm_ value of 40. Crystals were grown at 4 °C in 10% w/v PEG 8 K (89510, Sigma), 0.1 M imidazole pH 8 and 0.2 M calcium acetate (C1060, Sigma), and cryoprotected with 25% ethylene glycol (324558, Sigma). X-ray data were collected at the SOLEIL synchrotron on beamlines PX1 and PX2.

### X-ray data collection, processing, model building, and refinement

Diffraction data collection and refinement statistics are given in Supplementary Table 1. Crystallographic data were collected on the PROXIMA-1^52^ beamlines at Synchrotron SOLEIL (Saint-Aubin, France) and processed with XDS^53^ through XDSME (https://github.com/legrandp/xdsme)^54^. The strong diffraction anisotropy was corrected using the STARANISO program^55^. Initial phases for the apo-PabRPA crystal structure were obtained by Zn-SAD. The diffraction data from 3 crystals collected at the peak absorption wavelength of zinc K-edge (1.2827 Å) were merged to obtain a highly redundant dataset (>60). A unique zinc site was found using the SHELXC/D^56^ from which phases were calculated with PHASER^57^ and then improved by density modification with the PARROT program^58^. Thus, a first experimental electron density map at 4.5 Å resolution could be obtained in which an initial model could be assembled and completed using COOT^59^ combining the N-terminal region of Rpa1 previously determined and other partial models like the zinc finger. Later, this initial model could be improved with the help of AlphaFold^60^ predictions, and refined using a dataset reaching 3.7 Å in the best diffracting direction. The procedure proposed by Terwilliger^61^ was exploited to iteratively improved AlphaFold models using experimental information. Crystal structures of the PabRpa1 AROD-OB-1 module and the PabRPA Tri-C/d20T complex were determined by molecular replacement with MOLREP^62^ using the previously determined apo structure of PabRPA. All refinements were conducted with the BUSTER program^63^ using TLS motion groups and COOT was used for model reconstruction. In the case of PabRPA-apo and PabRPA Tri-C/d20T, local structure similarity restraints (LSSR) were used using hybrid target models constructed from a mix of higher resolution structures from the present work and AlphaFold-predicted models^60,61^. Details for all datasets are summarized in Supplementary Table 1.

### AlphaFold model predictions

AlphaFold model predictions were calculated either using the Google Colab platform and AlphaFold2_advanced form developed by the ColabFold team^64^ (https://colab.research.google.com/github/sokrypton/ColabFold) or using a local installation of ColabFold obtained from the LocalColabFold Github repository (https://github.com/YoshitakaMo/localcolabfold).

### Cryo-EM sample preparation

The PabRPA/d100T complex was obtained by mixing a poly-dT100 ssDNA (Eurogentec) with a 5-fold excess of PabRPA. The complex was then injected on a superdex 200 10/300 in buffer F (0.02 M HEPES pH 8, 0.1 M NaCl, and 0.002 M magnesium acetate (M5561, Sigma)). 3 µL of the eluted fraction (OD_280nm_ = 1.5) were applied to glow-discharged quantifoil R2/2 200 copper mesh grids (Electron Microscopy Sciences). After 20 seconds of pre-incubation, the grids were blotted for 6 seconds (force 0) using a Vitrobot Mark IV (ThermoFischer) at 100% humidity and 25 °C. For the PabRPA tetrameric supercomplex, 3 µL of purified PabRPA at 0.5 mg/mL were applied to glow-discharged Quantifoil R2/2 300 gold mesh grids. The grids were then blotted for 6 seconds (force 0).

### Cryo-EM data acquisition and image processing

The condensed, relaxed, and individual PabRPA/ssDNA complexes were obtained from two independent datasets collected on the same PabRPA/d100T sample. The dataset corresponding to the condensed PabRPA/d100T complex was collected on a 200 kV Glacios electron microscope (Thermo Fisher) equipped with a Falcon 3 detector operating in integration mode. The dataset corresponding to the relaxed PabRPA/d100T complex was collected on a 200 kV Glacios electron microscope equipped with a Falcon 4i detector and a bio-quantum energy filter (Gatan) operating in electron counting mode and was used to generate both relaxed and individual PabRPA/ssDNA complexes maps. Moreover, the PabRPA tetrameric supercomplex dataset was collected on a 300 kV Titan Krios electron microscope (Thermo Fisher) equipped with a K3 detector and a bio-quantum energy filter (Gatan). Motion correction and CTF estimation of the acquired movies were carried out in Cryosparc v4.1.0^65^. Image processing pipelines for each map are shown in Supplementary Fig. 5 and 9.

#### Procedure for the condensed PabRPA/d100T complex

Initial templates were generated with blob picking, and then used for template picking. Multiple initial models were generated, and the one that showed the interaction between two RPA heterotrimers was chosen and refined through heterogeneous and non-uniform refinement, achieving 8.2 Å resolution (Supplementary Fig. 5).

#### Procedure for the relaxed PabRPA/d100T complex

Initial templates were generated with blob picking, and then used for template picking. Several rounds of 2D-classifications were performed to isolate particles that showed only one RPA and used to generate three initial models of individual RPA. The best was chosen and refined through non-uniform refinement, achieving 3.9 Å resolution. Moreover, the particles showing the interaction between two RPA heterotrimers were used to generate an initial model that was refined through non-uniform refinement, achieving 8.0 Å resolution (Supplementary Fig. 5).

#### Procedure for the PabRPA tetrameric supercomplex cryo-EM structure

Initial 2D templates of the PabRPA tetrameric supercomplexes datasets were generated via blob picking and used to train a Topaz model^66^. Topaz picks were then used to generate three initial models, which were refined using heterogeneous refinement. 3D classification yielded a map containing AROD-OB-1, which was refined using non-uniform refinement and locally filtered with a lanczos filter, achieving 3.8 Å resolution. A high-resolution map that did not include the AROD-OB-1 module was refined with non-uniform refinement and C2 symmetry to achieve 3.4 Å resolution (Supplementary Fig. 9). Details for all datasets are summarized in Supplementary Table 2. 3D FSC is shown in Supplementary Fig. 14.

### Building and refinement of cryo-EM models

#### Procedure for the PabRPA tetrameric supercomplex cryo-EM structure

Four identical models of Pab-RPA Tri-C complex (PDBid: 8AAS) were manually placed and rigid-body fitted in the cryo-EM map using coot. The initial model was then subjected to global real-space refinement program from Phenix using secondary structure restraints. The refined model was further manually inspected and adjusted in coot.

#### Procedure for the PabRPA tetrameric supercomplex cryo-EM structure with the AROD-OB-1 module connecting two PabRPA molecules

The model was built by using the same procedure as for the PabRPA tetrameric complex (see above). In addition, Rpa1-AROD and OB-1 domains crystal structures (PDBid: 8AA9) were manually placed and rigid-body fitted using coot and Phenix. Procedure for the relaxed and condensed ssDNA-bound PabRPA complexes: The models of the ssDNA-bound Pab-RPA Tri-C complex (PDBid: 8AAS), and the structure of Rpa1 OB-1 (PDBid: 8AA9) were manually placed and rigid-body fitted using coot. B-factors were arbitrarily fixed constant at a value of 200. Refinement details for all datasets are summarized in Supplementary Table 2.

### Structure analysis

Electrostatic calculations were performed by using APBS^67^. Interaction surfaces were calculated by using the PISA webserver^68^. Surface conservations were calculated using CONSURF^69^. Structure comparisons were performed by using DALI^70^. All figures were prepared using UCSF Chimera X^71^.

### Negative-staining microscopy

The PabRPA/pM13 complex was reconstituted by mixing 1 µL of M13mp18 ssDNA (New England Biolabs) at 250 µg/mL with 1 µL of PabRPA at 30 µM (200× excess) in 98 µL of buffer F. 3 µL of the mixture was deposited on glow-discharged carbon-coated copper grids CF400-CU (Electron Microscopy Sciences) and contrasted 3 × 1 minute in 2% uranyl acetate. Data collection was performed using a Tecnai biotwin T12 (Thermo Fisher) equipped with a LaB6 filament, operating at 120 keV. Images were recorded using an Eagle camera (Thermo Fisher) at a nominal magnification of 49,000, using a 3 µm defocus.

### Electromobility shift assay

2.5 pmol of 3’-FAM ssDNA-24-mer (5’-GCCTGCAGGTCGACTCTAGAGGAT-3’) or dsDNA-24-mer were mixed with increasing amounts of *Pab*RPA in Tris-HCl 30 mM pH 7.5, NaCl 300 mM, 0.5 g/L bovine serum albumin (BSA), 5% Ficoll (20 µL final volume). Reactions were carried out to reach equilibrium at 60 °C for 1 h before migration in 1% agarose gel at 4 °C under native conditions (90 V, 30 mA). Images were acquired with Typhoon9400 (GE Healthcare).

### Surface plasmon resonance (SPR)

Surface plasmon resonance data were acquired on a Reichert SR7000DC spectrometer equipped with a self-sampling Reichert SR7100 injection system. Sensorchips consisted of a glass slide coated with a gold film, the latter being already functionalized by a mixed self-assembled monolayer (SAM) of 90% dithiol aromatic PEG6-COOH and 10% of dithiol aromatic PEG3-COOH. Neutravidin was immobilized on the SAM, in both flow cells, using classical EDC:NHS coupling chemistry. Appropriate amounts of DNA were immobilized onto surfaces by successive injections at 25 µL/min of 1 µM of 5’-biotin-TEG labeled 32mer ssDNA (5’-TGCCAAGCTTGCATGCCTGCAGGTCGACTCTA-3’), ssRNA (5’-UGCCAAGCUUGCAUGCCUGCAGGUCGACUCUA-3’) or dsDNA (Eurogentec) diluted in buffer E supplemented with 0.05% tween. Binding assays were performed at 25 °C by injecting increasing concentrations of protein solutions for 300 s at 25 µL/min. The raw sensograms were processed by double referencing, i.e., subtracting both the signals measured on the reference flow cell and the signals measured for blank injections. Resulting sensorgrams were analyzed by Scrubber2.0 (BiolLogic) following several Monte Carlo fits without mass transport limitation, assuming a single equilibrium system for PabRPA, Rpa2, Rpa3, and Rpa1/Rpa2 and a two-step equilibrium system for Rpa1.

### Biolayer interferometry (BLI)

ssDNA-protein binding assays were performed on an Octet RED384 BLI instrument (ForteBio). 3’-biotin-TEG labeled poly-dT35 ssDNA was captured at a concentration of 5 µg/mL for 100 s on streptavidin sensors. For protein-protein binding assays, His-tagged proteins were captured at a concentration of 5 µg/mL for 100 s on NTA sensors, subtracting non-specific signals from reference sensors loaded with His-tagged maltose-binding protein (MBP). Each binding experiment was performed at least three times at 25 °C in buffer E supplemented with 0.2 mg/mL BSA (A2153, Sigma). Data were analyzed with Prism 9.4. A blank sample reference with buffer-only was subtracted from all curves. Affinities were determined by fitting the concentration dependence of the experimental steady-state signals, using the following equation Req = Rmax * [RPA]/(Kd + [RPA]).

### Mass photometry (MP)

The MP experiments were performed at room temperature using the 2MP instrument (Refeyn, Oxford, UK). The 24 × 50 mm microscope coverslips (CG15KH, Thorlabs) were prepared by cleaning with MilliQ water and isopropanol (w/I/w/I/w), and drying under a stream of filtered air. A piece of clean, precut 2 × 2-well culture well gasket (GBL103250, Sigma) was attached to the coverslip. 18 µl of the filtered buffer were loaded into a well of the culture well gasket, and, after MP focusing with immersion oil (ref 518 F, Zeiss), 2 µl of 10X sample were added into the same well. Immediately after the solution was mixed by pipetting, a 1 min video was recorded using the AcquireMP (Refeyn, Oxford, UK) software. MP video files were processed using the DiscoverMP software (Refeyn, Oxford, UK). The threshold parameter value of five was used in the analysis. MP contrast distributions were plotted as histograms. For all plots, the histogram bin size was set to 0.002 contrast units. To obtain information on the contrast distribution species, the MP histograms were fit with Gaussian peaks. For each fitted species, the best fit Gaussian peak position and area represent their average contrast value and their number fraction, respectively. The molecular mass of the protein was estimated from the MP contrast distribution by applying the calibration obtained using BSA (A7638, Sigma) and urease (U7752, Sigma).

### Small-angle X-ray scattering (SAXS)

SAXS data of the PabRPA ΔWH complex (50 μl at 1 mg/ml) in buffer E was collected in batch Mode at beamline SWING (synchrotron SOLEIL). The curves were background-subtracted using FOXTROT and analyzed using the ATSAS 3.0.2 software suite. The normalized Kratky was calculated according to^72,73^ using the value determined by Guinier analysis. P(r) functions were computed from the scattering curves by an indirect transform method in GNOM^74^.

### Molecular mass measurements by size exclusion chromatography coupled to static light scattering detection (SEC-SLS) and viscometry

The oligomerization states of PabRPA ΔWH, PabRPA Tri-C, or RPA from *T.nautili* at 20 µM in presence or absence of 24 µM poly-dT25 ssDNA (Eurogentec) were determined by size exclusion chromatography coupled to a triple detection (concentration detector: UV detector, refractometer; static light scattering 7°, 90°; viscometer) on a Omnisec resolve/reveal instrument (Malvern Panalytical). The column and detectors were equilibrated with the filtered and degazed running buffer E prior measurement. All proteins were injected (100 µl) and eluted at 0.4 ml/min on a Superdex 200 increase 10/300 GL column (Cytiva). Detections were performed at 20 °C or 65 °C. External calibration was done with BSA using an injection of 100 μl at 1.4 mg/ml. The refractive index, static light scattering, and viscosity measurements were processed to determine the mass average molecular mass and the intrinsic viscosity using the OMNISEC V11.10 software (Malvern Panalytical).

### Analytical ultracentrifugation assays

Sedimentation velocity experiments were performed with on a Beckman-Coulter Optima analytical ultracentrifuge (Beckman-Coulter, USA) with an An-60 Ti rotor at 20 °C. Rpa1-AROD-OB-1 at a concentration of 6.5 mg/ml was centrifuged at 100,000 × *g* in 3-mm double-sector epoxy centerpieces. 100 scans were collected at 1 min intervals with a radial step size of 0.001 cm. Detection of the protein complex as a function of radial position and time was performed by absorbance measurements at 250 nm, 280 nm, and by interference detection. Profiles were analyzed using the continuous (s) distribution model of the software Sedfit^75^. The partial specific volume of the protein, 0.746 was theoretically calculated in Sedfit. The buffer viscosity of 0.01031 Poise and the buffer density of 1.00620 were respectively determined with the Viscosizer TD (Malvern Panalytical, UK) and the DMA 5000 M (Anton Paar).

### Reporting summary

Further information on research design is available in the Nature Portfolio Reporting Summary linked to this article.

## Supplementary information

Supplementary Information

Peer Review File

Description of Additional Supplementary Files

Supplementary Movie 1

Supplementary Movie 2

Reporting Summary

## Data Availability

The PabRpa1 AROD-OB-1 domains, PabRPA, and PabRPA Tri-C/d20T crystal structures were deposited in the Protein Data Bank with PDB identifier: 8AA9, 8AAJ, and 8AAS, respectively. The all-atom cryo-EM structure of PabRPA tetramer, as well as the 3D-class showing the AROD-OB-1 domains are deposited and are available at the Protein Data Bank (PDB) and at the Electron Microscopy Data Bank (EMDB) with the identifier 8C5Y and EMDB-16444, 8C5Z, and EMDB-16445, respectively. The cryo-EM maps of the condensed PabRPA/ssDNA complex can be found with the identifiers 8OEL and EMDB-16827 and for the relaxed PabRPA/ssDNA complex with the identifier 8OEJ and EMDB-16826. The individual PabRPA on ssDNA is deposited in the EMDataResource with the identifier EMDB-16448 respectively. Source data are provided with this paper.

## Source data

Source Data
