# Supplementary material for: DNA-binding mechanism and evolution of replication protein A
Source: Nat Commun. 2023 Apr 22;14:2326. doi: 10.1038/s41467-023-38048-w (PMC10122647; doi:10.1038/s41467-023-38048-w)
Supplement: Supplementary file 1 — Supplementary Information [file 41467_2023_38048_MOESM1_ESM.pdf]

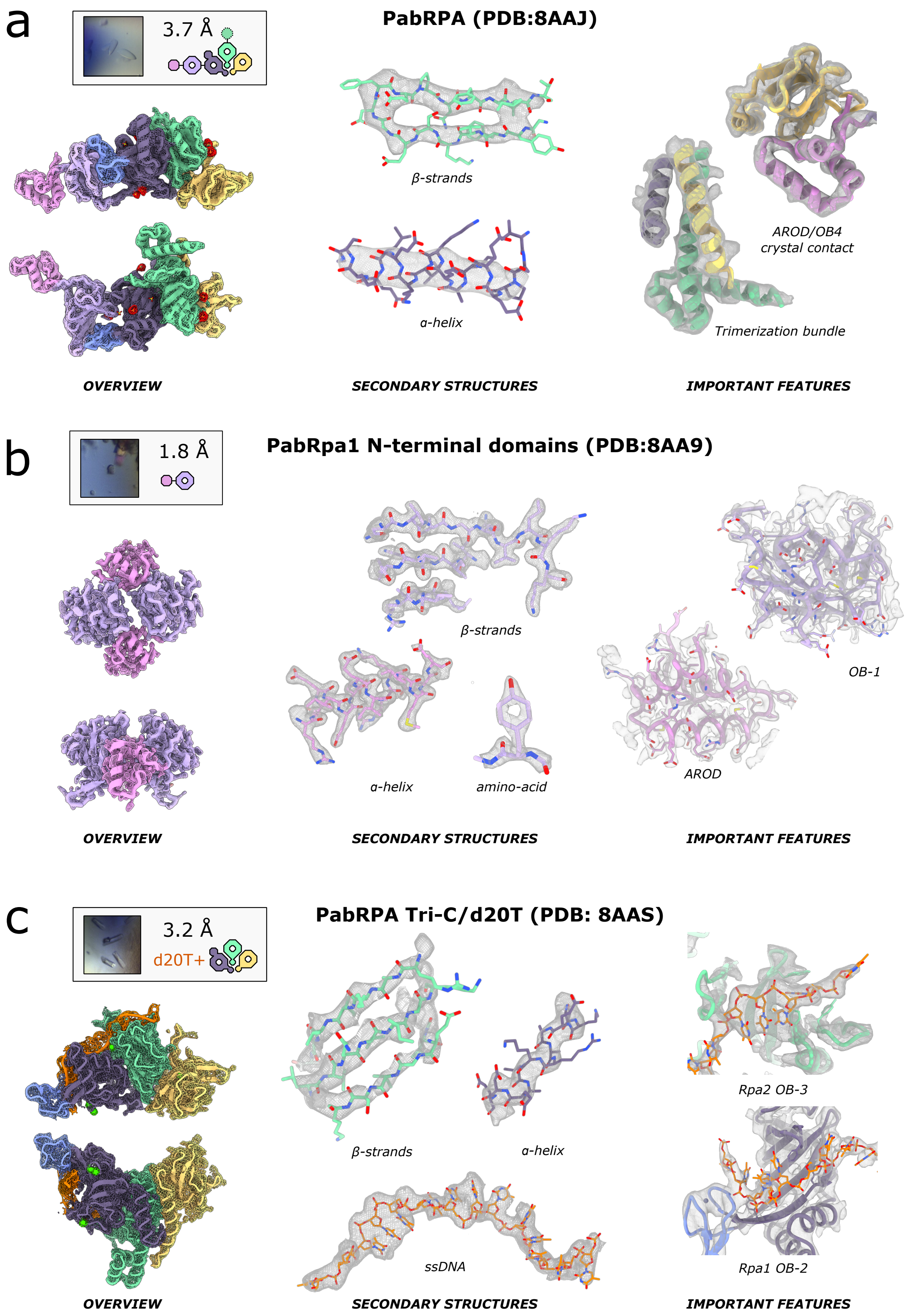

Supplementary figure 1: Electron density in representative regions of the PabRPA crystal structures. PabRPA (a), PabRPA Rpa1 N-terminal domains (b) and PabRPA Tri-C/20dT (c) crystal structures are shown in cartoon representations in the left panels. Right panels show close views of functionally important regions of PabRPA with side chains atoms represented as sticks. The gray mesh is the maximum likelihood 2mFo-DFc electron density map contoured at a level of 1 σ.

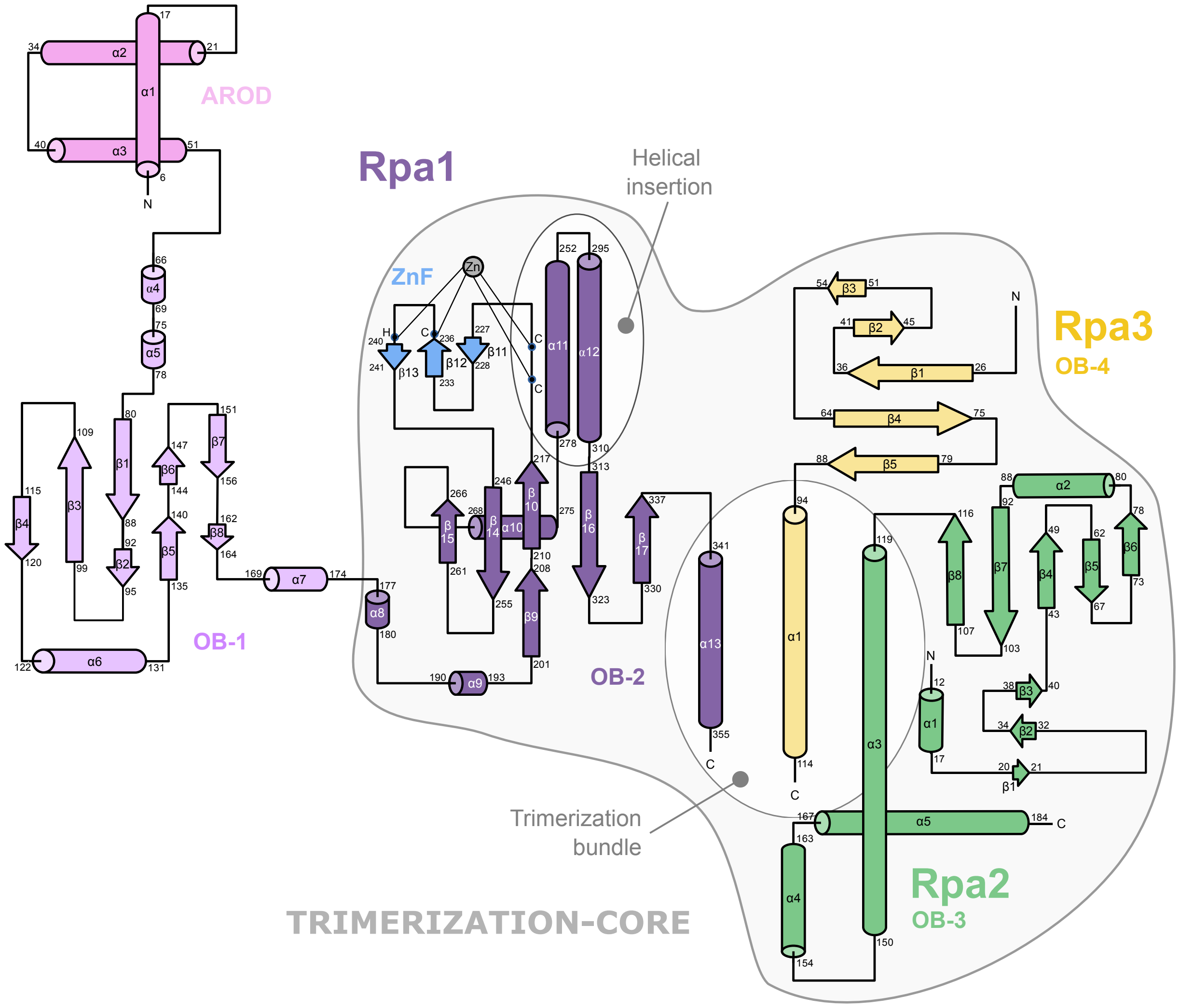

Supplementary figure 2: Topological diagram of PabRPA. Two-dimensional diagram of protein topology for three subunits of PabRPA.

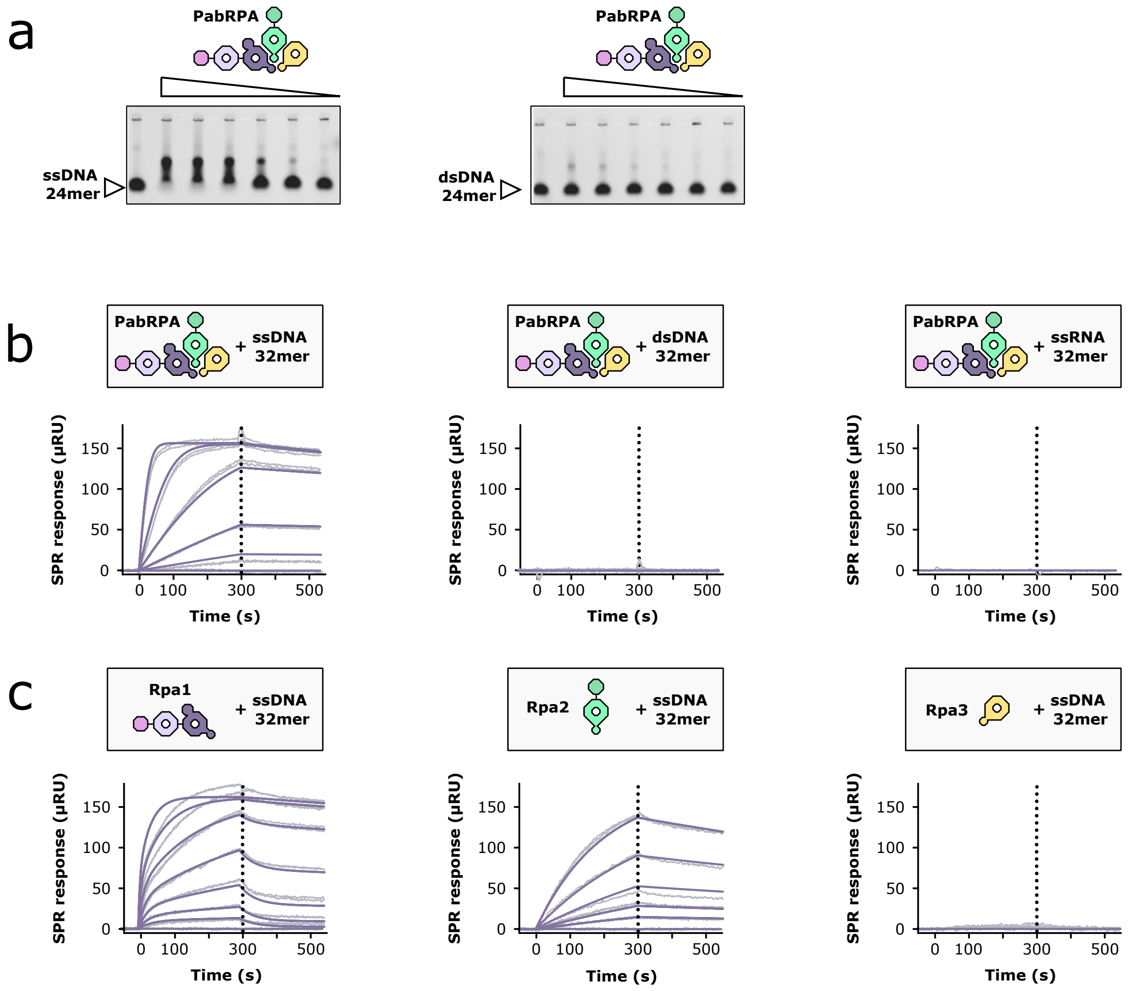

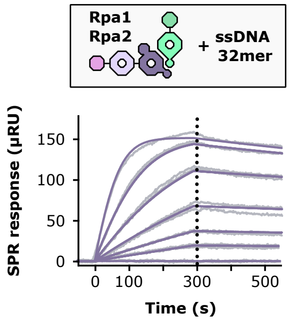

d

	PabRPA	Rpa1 / Rpa2	Rpa1	Rpa2	Rpa3
**K_D (M)**	$(710 \pm 10) \times 10^{-12}$	$(1.23 \pm 1) \times 10^{-9}$	$(82 \pm 2) \times 10^{-9}$ [a]<br>$(4.7 \pm 1) \times 10^{-9}$ [b]	$(31.4 \pm 3) \times 10^{-9}$	> 10 μM
**k_a ($M^{-1}.s^{-1}$)**	$(4,26 \pm 2) \times 10^5$	$(2.80 \pm 2) \times 10^5$	$(2.74 \pm 2) \times 10^5$ [a]<br>$(4.14 \pm 4) \times 10^4$ [b]	$(2.55 \pm 1) \times 10^4$	*N.D.*
**k_d (s^{-1})**	$(3.02 \pm 6) \times 10^{-4}$	$(3.44 \pm 4) \times 10^{-4}$	$(2.30 \pm 7) \times 10^{-2}$ [a]<br>$(1.95 \pm 7) \times 10^{-4}$ [b]	$(8.0 \pm 1) \times 10^{-4}$	*N.D.*

Supplementary figure 3: Nucleic-acids binding properties of PabRPA. a. Electrophoretic mobility shift assay of ssDNA-24mer and dsDNA-24mer (2.5 pmol) in presence of decreasing concentrations of PabRPA (312, 62, 12, 2.5, 0.5, 0.1 pmol). b. PabRPA binding specificity on nucleic acids. Specific binding of PabRPA heterotrimer (5, 2.5, 1.25, 0.63, 0.32, 0.16 nM, n=2) on immobilized ssDNA-32mer (left), dsDNA-32mer (middle) and ssRNA-32mer measured by surface plasmon resonance (RU: resonance units). c. Role of PabRPA subunits on ssDNA binding. Specific binding of Rpa1 (1000, 500, 250, 125, 63, 31, 16 nM, n=2), Rpa2 (500, 250, 125, 63, 31, 16 nM, n=2), Rpa3 (1000, 500, 250 nM, n=2), or Rpa1/Rpa2 complex (1000, 500, 250, 125, 63, 31, 16 nM, n=2) on immobilized ssDNA-32mer measured by surface plasmon resonance. d. Kinetic rate constants (ka and kd) and equilibrium dissociation constants (KD) for the binding interactions of PabRPA constructs on ssDNA-32-mer.

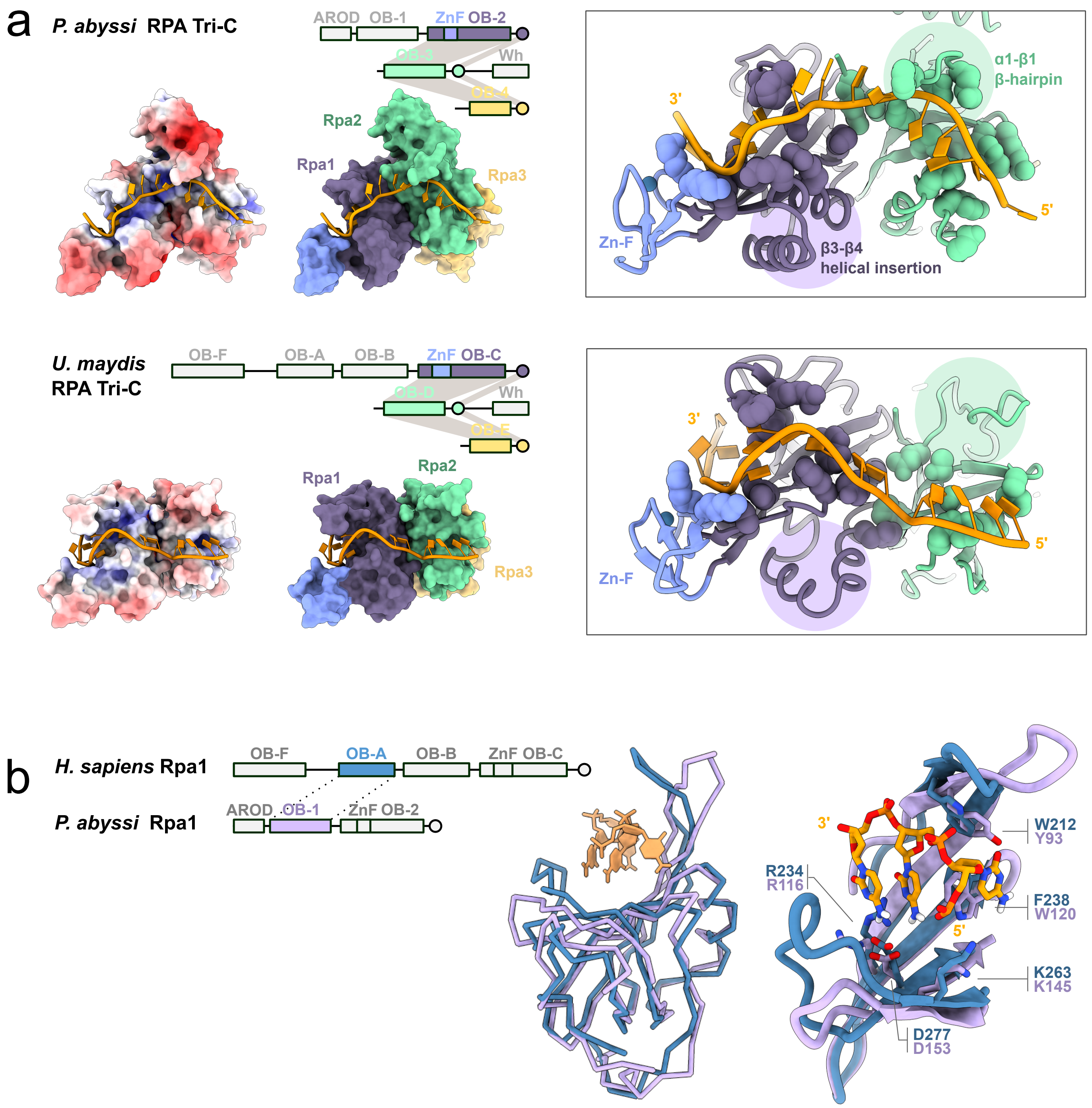

Supplementary figure 4: Comparison of archaeal and eukaryotic RPA ssDNA binding determinants. a. Comparison of DNA-bound P. abyssi and U. maydis (4GOP) Tri-C structures with focused views on Rpa1 (purple and blue) and Rpa2 (green). ssDNA-contacting residues are shown as spheres. b. Superimposition of the P. abyssi Rpa1 OB-1 (in purple) with the H. sapiens Rpa1 OB-A (in blue) (1JMC) crystal structures reveals conserved ssDNA-binding amino-acids.

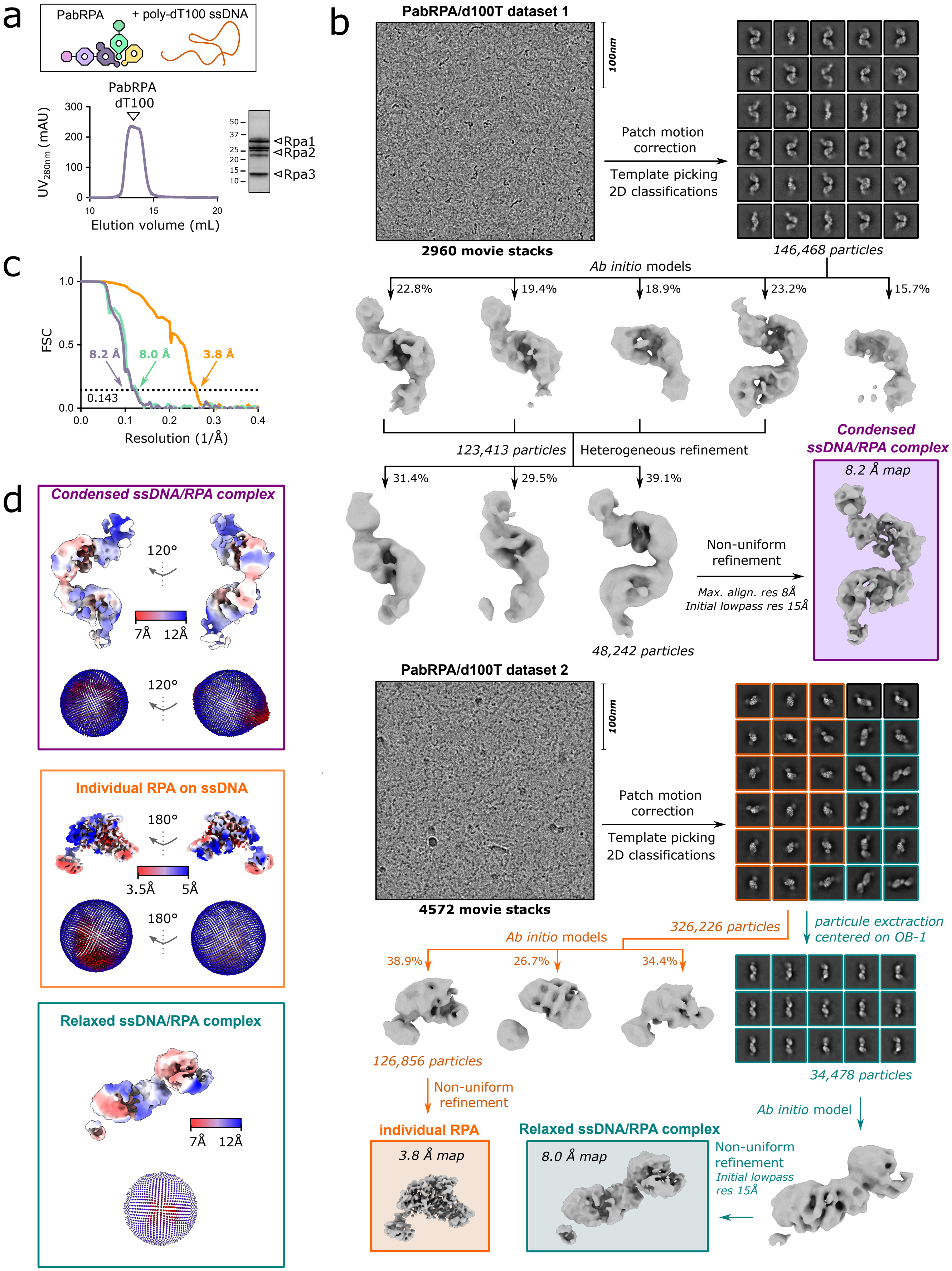

Supplementary figure 5: Cryo-EM structure determination of PabRPA nucleofilaments. a. Size-exclusion chromatography (SEC) profile (left) and SDS-PAGE analysis (right) of PabRPA bound to a poly-dT100 ssDNA b. Data processing workflow (detailed in methods). Two independent cryo-EM datasets showing condensed (on top) and relaxed (on bottom) ssDNA/PabRPA complexes were collected. c. Resolution of final reconstructions determined by gold standard FSC at the 0.143 criterion. d. Local resolution evaluations of the final reconstructions (left) and Euler angle distributions of particles used for the final reconstructions (right).

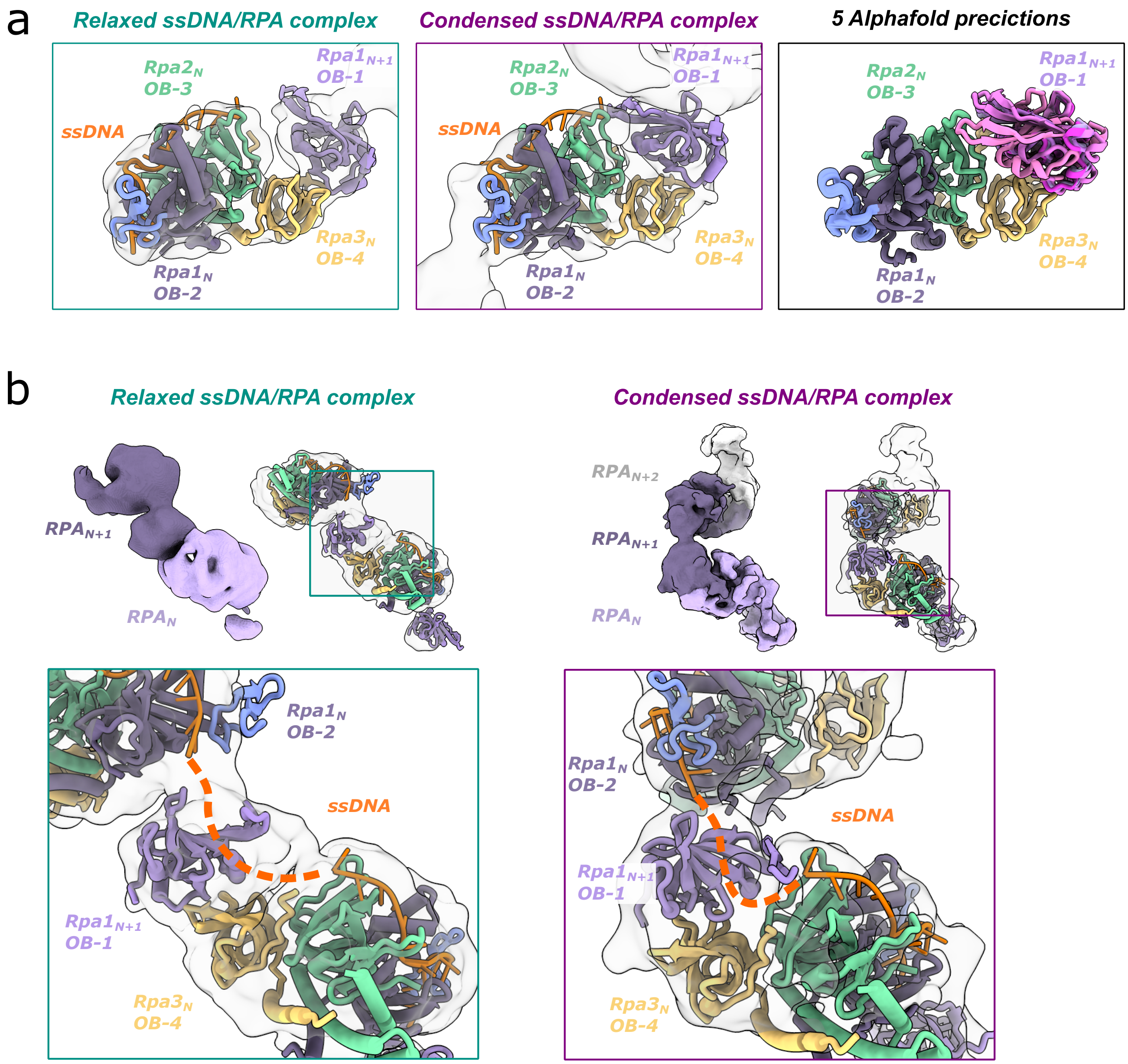

Supplementary figure 6: the OB1/OB4 contact facilitates the passage of ssDNA from one RPA to the other. a. Comparatives views of the OB1/OB4 interaction in the relaxed ssDNA/RPA complex (left), condensed ssDNA/RPA complex, and in Alphafold2 predictions. All the structures are superimposed on Rpa3 subunits. b. In both relaxed and condensed complexes, the DNA-binding groove of OB-1 is ideally located to route the ssDNA from a Tri-C to the other.

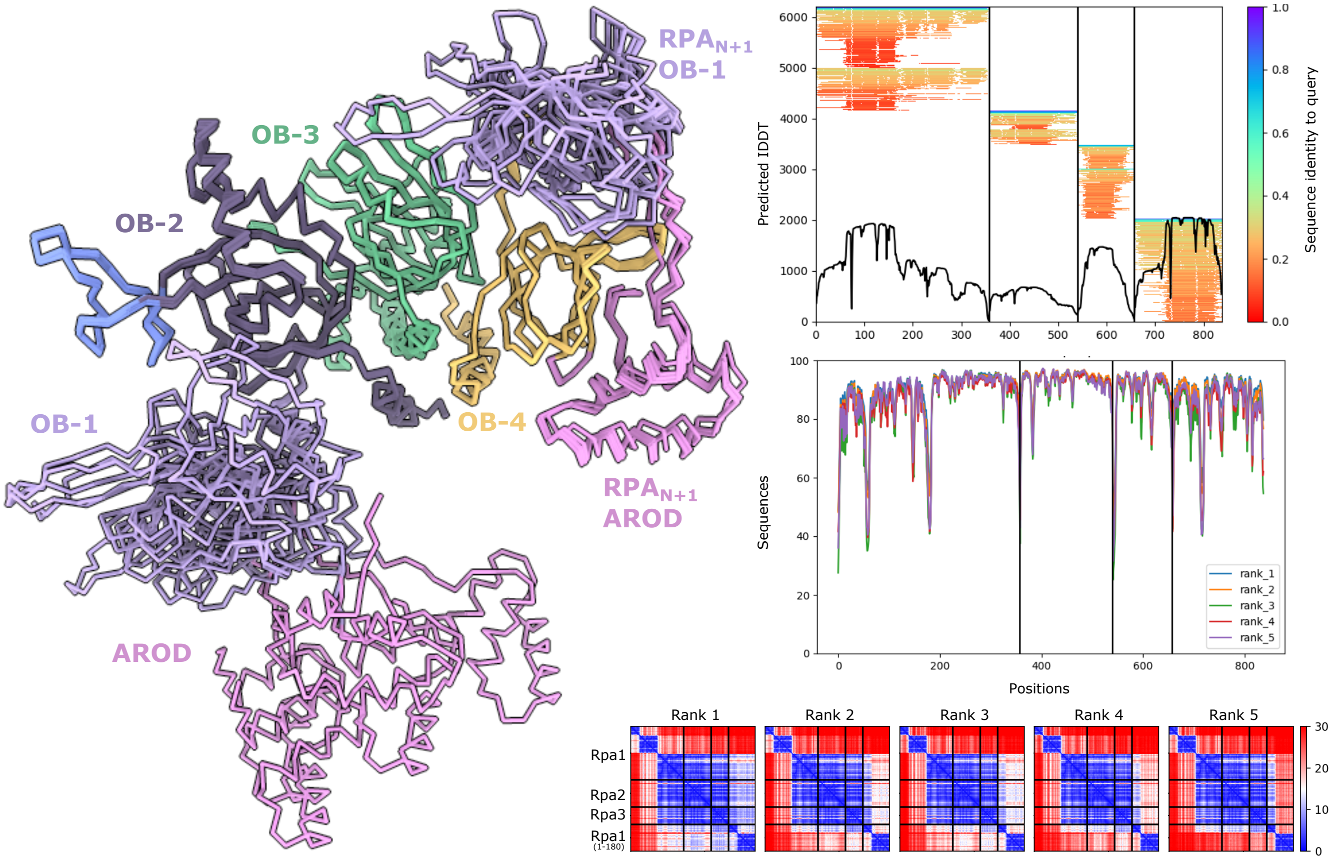

Supplementary figure 7: AlphaFold prediction of the interface between two adjacent PabRPAs. Left: superposition of the five AlphaFold-Multimer predicted models (Evans et al., 2021) for the interaction of one PabRPA-ΔWH heterotrimer with two first domains of PabRPA1 (AROD-OB1, 1-180). Right: AlphaFold generated plots of MSA sequence coverage (top), of predicted LDDT vs position along the protein sequence (middle) and of predicted aligned error (bottom, where each protein chain of the complexe is plotted against itself and against the other chain).

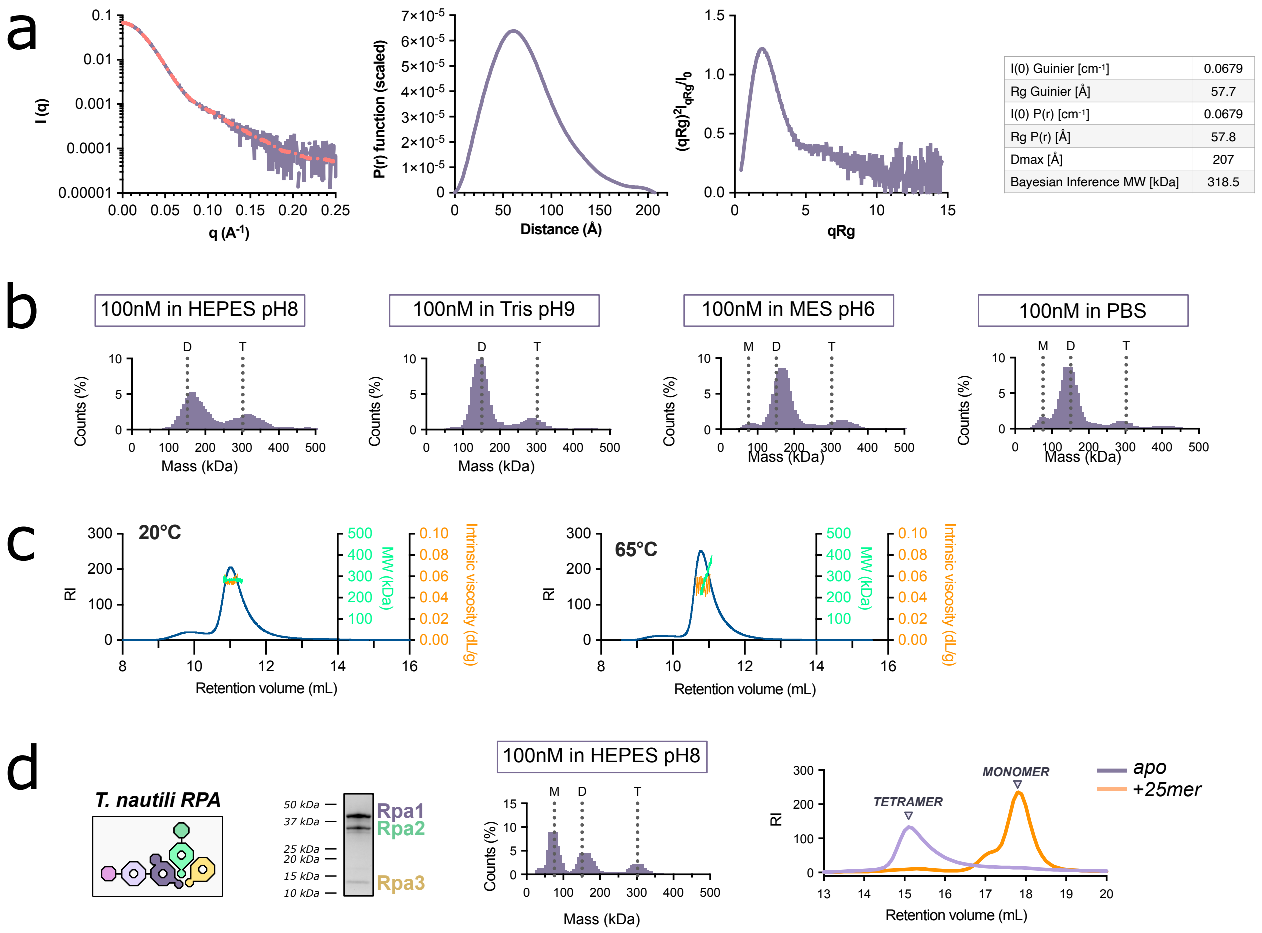

Supplementary figure 8: Archaeal RPA oligomerizes in solution. a. In solution study of PabRPA structure by SAXS. On top left, SAXS profile of PabRPA (in purple) with superimposition of fitted scattering pattern that allowed the generation of the distance distribution function P(r) (red dotted line). On right, distance distribution functions P(r) derived from the scattering patterns of PabRPA obtained using the program GNOM. On bottom left, dimensionless Kratky plot scattering. Scattering derived parameters are provided in the table. b. Characterization of PabRPA oligomeric states at different pH by mass photometry. Expected molecular weights for monomeric (M), dimeric (D) and tetrameric (T) PabRPA super-complexes are indicated with dotted lines c. PabRPA molecular mass (Green) and intrinsic viscosity (orange) measurements by SEC-SLS coupled with an inline viscometer. Measurements were performed at 20°C (higher panel) and 65°C (lower panel). d. Characterization of Thermococcus nautili's RPA oligomeric states in solution. On left panel, SDS-PAGE analysis of the RPA heterotrimer from T. nautili. This analysis was repeated two times independently. On middle panel, oligomeric states at 100 μM characterized by mass photometry. On right panel, SEC-SLS characterization in presence or absence of poly-d25T ssDNA.

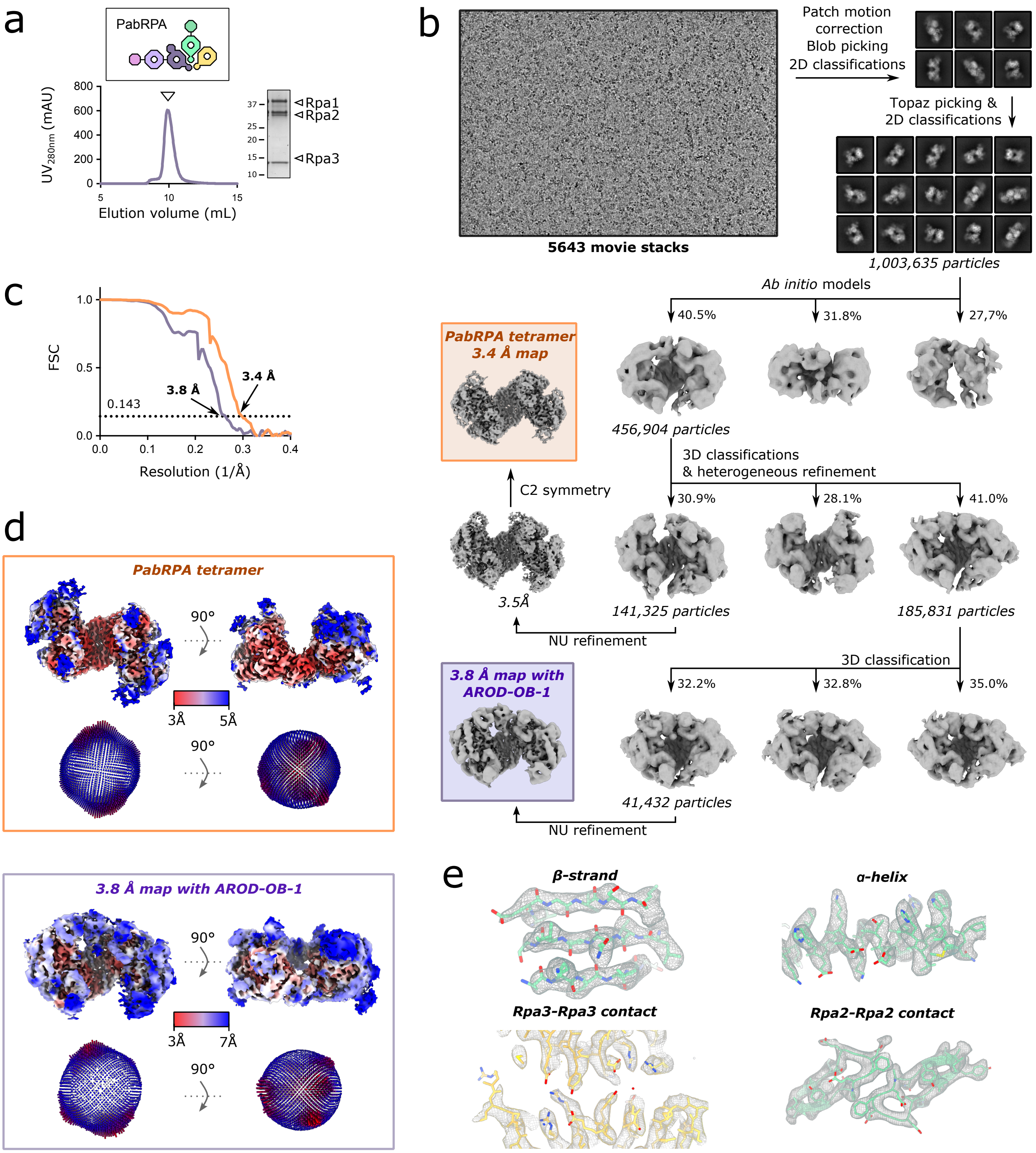

Supplementary figure 9: Cryo-EM structure determination of the tetrameric RPA super-structure. a. Size-exclusion chromatography (SEC) profile (left) and SDS-PAGE analysis (right) of PabRPA. This analysis was repeated at least three times. b. Data processing workflow (detailed in methods). c. Resolution of final reconstructions determined by gold standard FSC at the 0.143 criterion. d. Local resolution evaluations of the final reconstructions (top) and Euler angle distributions of particles used for the final reconstructions (bottom). e. Representative views of the cryo-EM map showing secondary structure elements and key regions of the tetrameric RPA assembly.

a

	PabRPA	Rpa1-Nter	TriC/20mer	PabRPA/100mer	PabRPA tetramer
AROD/OB1	✓	✅	n/a		
AROD/OB4	✓	n/a	n/a		✅
OB1/OB4		n/a	n/a	✅	✅
OB3/OB3	✓	n/a			✅
OB4/OB4		n/a	✓		✅

✅ biological unit ✓ crystal contact

b

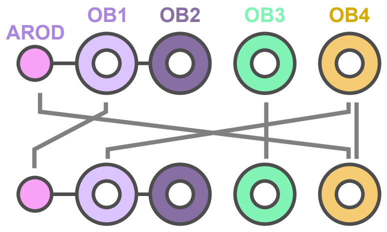

c

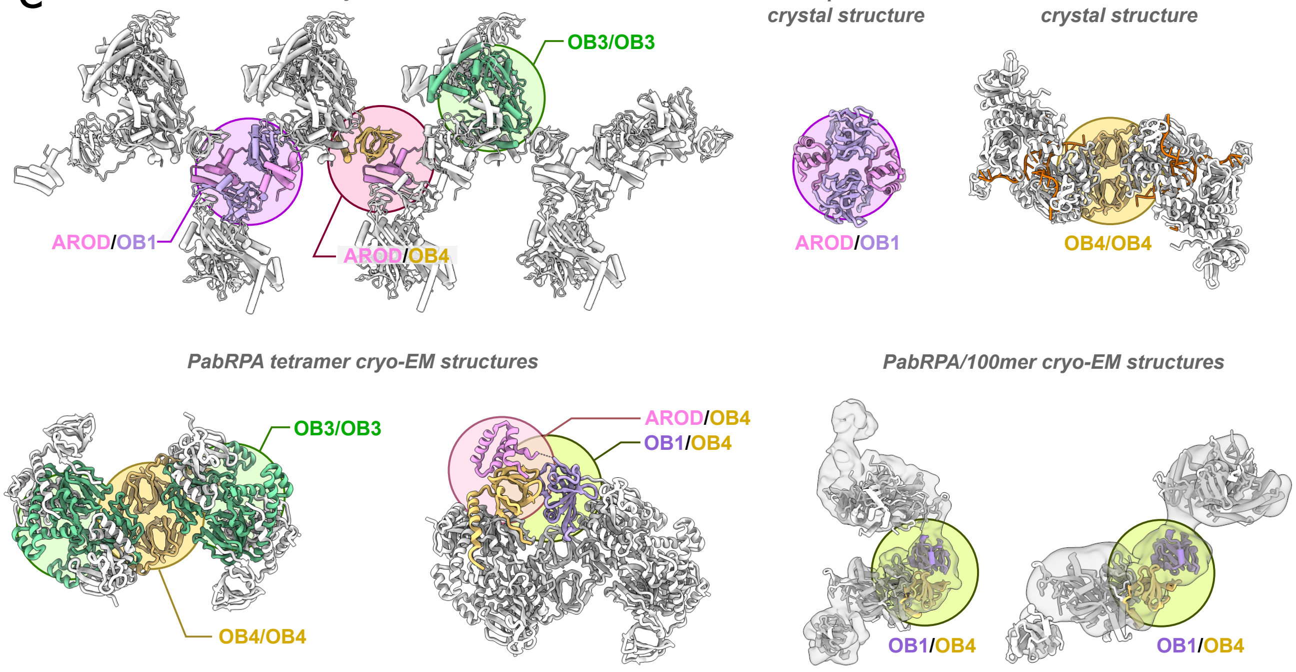

Supplementary figure 10: Multivalent interactions mediated by PabRPA OB-domains. Table (a), schematic representation (b) and structures (c) showing the different OB-domains interactions observed in this study.

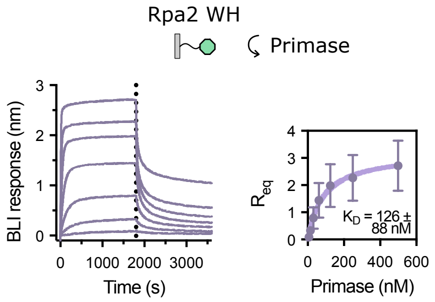

Supplementary figure 11: Rpa2 WH domain binds to the archaeal primase. Binding of primase (7.8, 15.6, 31.2, 62.5, 125, 250, 500 nM, n=3) to immobilized PabRpa2 WH C-terminal domain (180-269) measured by biolayer interferometry. Steady-state analysis was performed using the average signal measured at the end of the association steps. Data are presented as mean values +/- standard deviations.

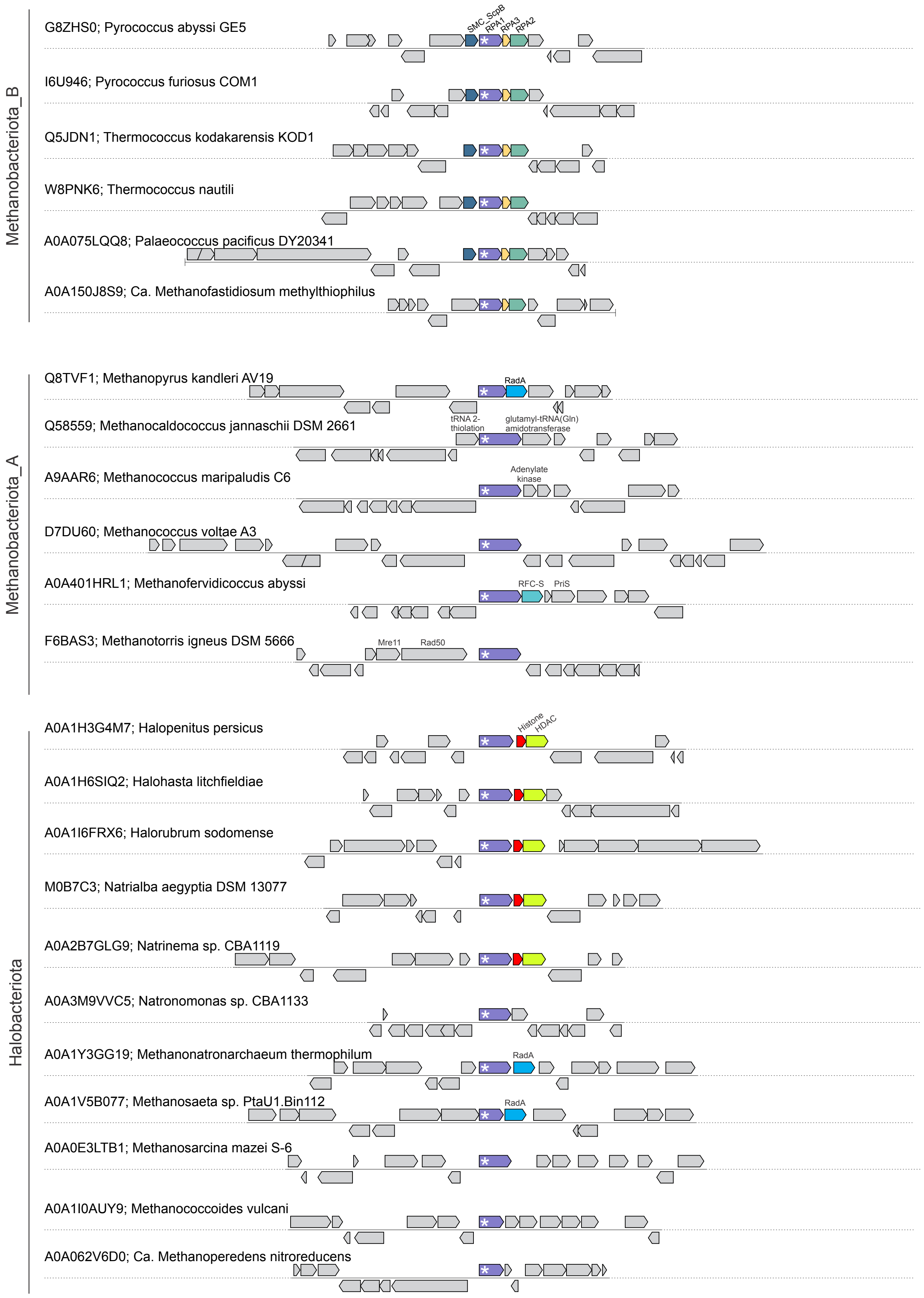

Methanobacteriota_B
G8ZHS0; Pyrococcus abyssi GE5
SMC_ScpB
RPA1
RPA3
RPA2
I6U946; Pyrococcus furiosus COM1
Q5JDN1; Thermococcus kodakarensis KOD1
W8PNK6; Thermococcus nautili
A0A075LQQ8; Palaeococcus pacificus DY20341
A0A150J8S9; Ca. Methanofastidiosum methylthiophilus
Methanobacteriota_A
Q8TVF1; Methanopyrus kandleri AV19
RadA
tRNA 2-thiolation
glutamyl-tRNA(Gln) amidotransferase
Q58559; Methanocaldococcus jannaschii DSM 2661
Adenylate kinase
A9AAR6; Methanococcus maripaludis C6
D7DU60; Methanococcus voltae A3
A0A401HRL1; Methanofervidicoccus abyssi
RFC-S
PriS
F6BAS3; Methanotorris igneus DSM 5666
Mre11
Rad50
Halobacteriota
A0A1H3G4M7; Halopenitus persicus
Histone
HDAC
A0A1H6SIQ2; Halohasta litchfieldiae
A0A1I6FRX6; Halorubrum sodomense
M0B7C3; Natrialba aegyptia DSM 13077
A0A2B7GLG9; Natrinema sp. CBA1119
A0A3M9VVC5; Natronomonas sp. CBA1133
A0A1Y3GG19; Methanonatronarchaeum thermophilum
RadA
A0A1V5B077; Methanosaeta sp. PtaU1.Bin112
RadA
A0A0E3LTB1; Methanosarcina mazei S-6
A0A1I0AUY9; Methanococcoides vulcani
A0A062V6D0; Ca. Methanoperedens nitroreducens

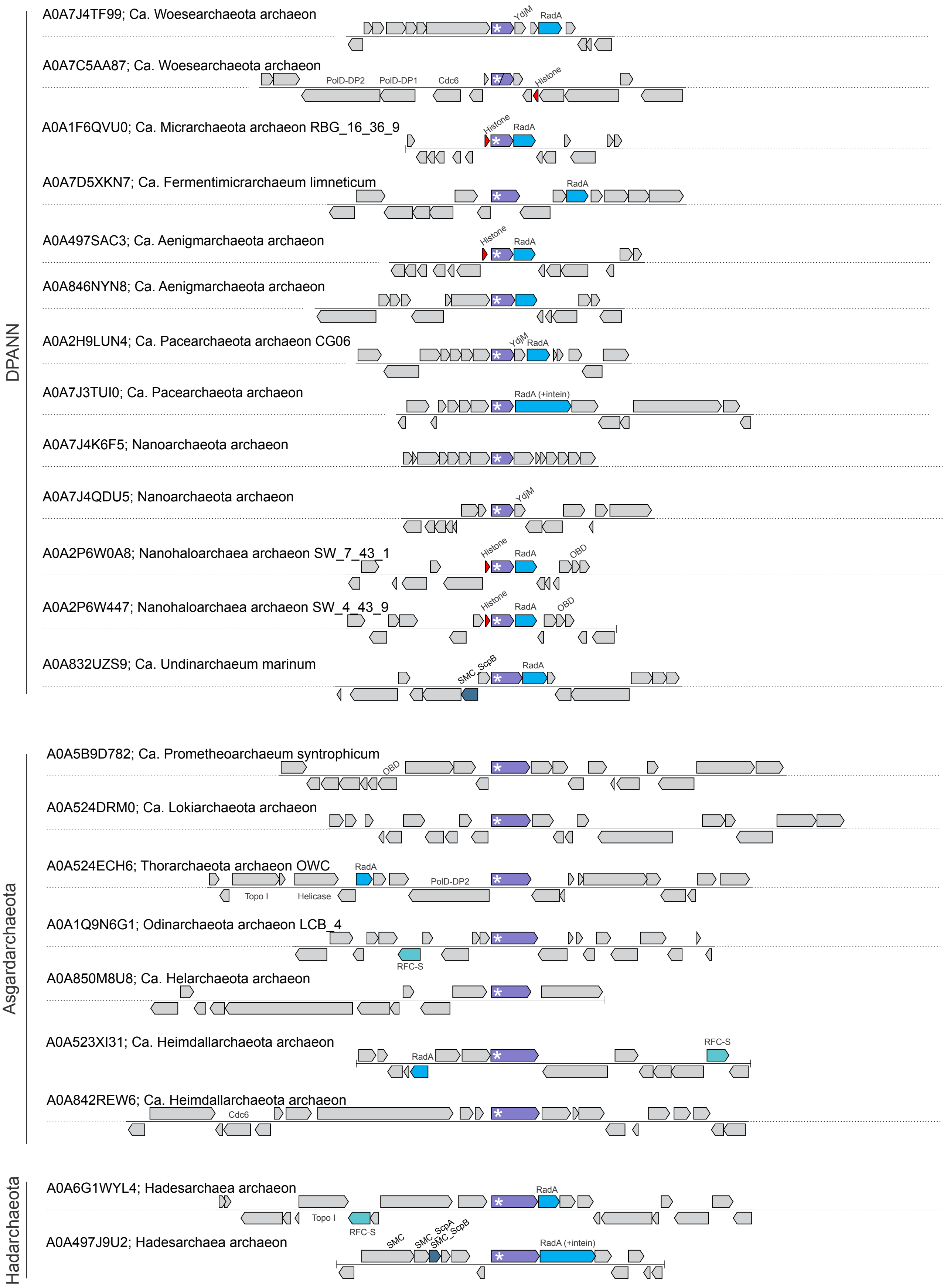
DPANN
A0A7J4TF99; Ca. Woesearchaeota archaeon
YdjM
RadA
A0A7C5AA87; Ca. Woesearchaeota archaeon
PolD-DP2
PolD-DP1
Cdc6
Histone
A0A1F6QVU0; Ca. Micrarchaeota archaeon RBG_16_36_9
Histone
RadA
A0A7D5XKN7; Ca. Fermentimicrarchaeum limneticum
RadA
A0A497SAC3; Ca. Aenigmarchaeota archaeon
Histone
RadA
A0A846NYN8; Ca. Aenigmarchaeota archaeon
A0A2H9LUN4; Ca. Pacearchaeota archaeon CG06
YdjM
RadA
A0A7J3TUI0; Ca. Pacearchaeota archaeon
RadA (+intein)
A0A7J4K6F5; Nanoarchaeota archaeon
A0A7J4QDU5; Nanoarchaeota archaeon
YdjM
A0A2P6W0A8; Nanohaloarchaea archaeon SW_7_43_1
Histone
RadA
OBD
A0A2P6W447; Nanohaloarchaea archaeon SW_4_43_9
Histone
RadA
OBD
A0A832UZS9; Ca. Undinarchaeum marinum
SMC_ScpB
RadA
Asgardarchaeota
A0A5B9D782; Ca. Prometheoarchaeum syntrophicum
OBD
A0A524DRM0; Ca. Lokiarchaeota archaeon
A0A524ECH6; Thorarchaeota archaeon OWC
RadA
PolD-DP2
Topo I
Helicase
A0A1Q9N6G1; Odinarchaeota archaeon LCB_4
RFC-S
A0A850M8U8; Ca. Helarchaeota archaeon
A0A523XI31; Ca. Heimdallarchaeota archaeon
RadA
RFC-S
A0A842REW6; Ca. Heimdallarchaeota archaeon
Cdc6
Hadarchaeota
A0A6G1WYL4; Hadesarchaea archaeon
RadA
Topo I
RFC-S
A0A497J9U2; Hadesarchaea archaeon
SMC
SMC_ScpA
SMC_ScpB
RadA (+intein)

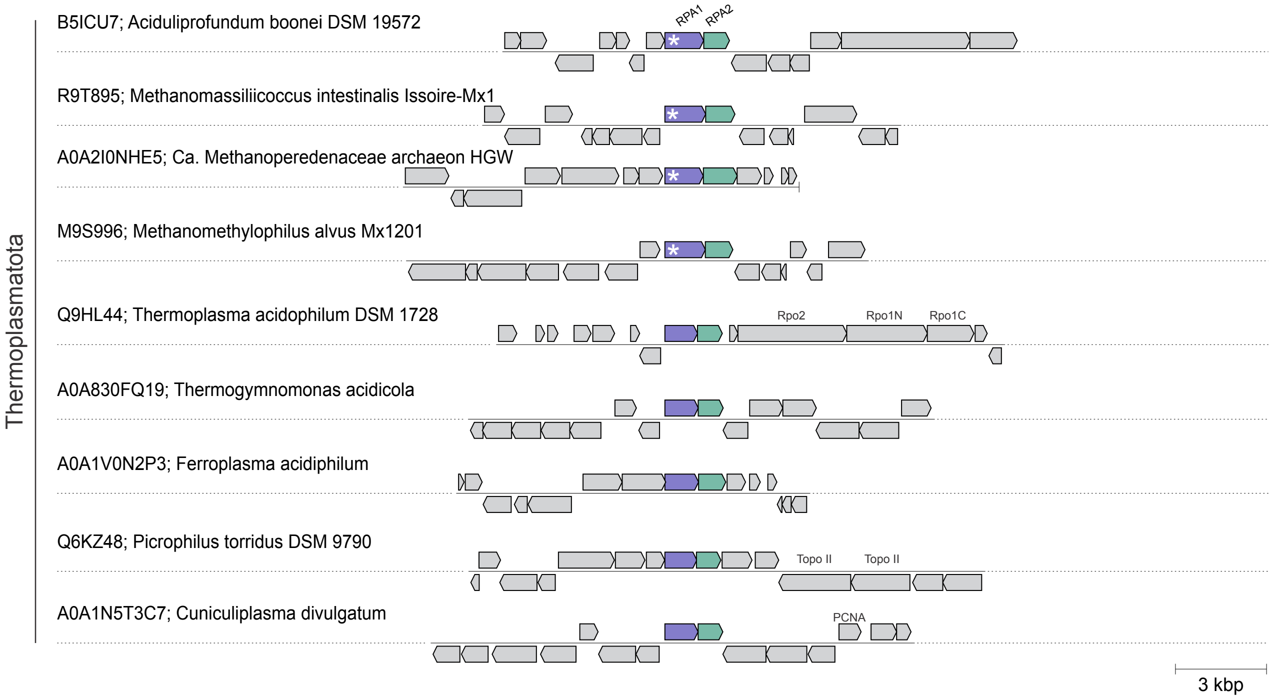

Supplementary figure 12: Comparison of genomic loci encoding Rpa1 genes in Archaea. Genomic organizations of the loci encoding Rpa1 genes are shown for representative species from each major archaeal phyla. Each locus is indicated with the UniProt accession number of the Rpa1 gene, followed by the species name. All loci are shown as -7 and +7 genes around Rpa1 gene. Rpa1 genes are embedded within highly variable genomic neighborhoods, which typically vary even for relatively closely related organisms. Presence of the Archaea-specific AROD is indicated with white asterisks. Strikingly, AROD is present in all Rpa1 sequences, except for the 5 Thermoplasmatales members. It is noteworthy that Methanomassiliicoccales within Thermoplasmatota do contain AROD, suggesting that AROD was lost within Thermoplasmatales following their divergence from a common ancestor with Methanomassiliicoccales. Interestingly, Aciduliprofundum boonei which is even closer to the Thermoplasmatales also contain AROD. DNA metabolism proteins encoding genes are represented in color.

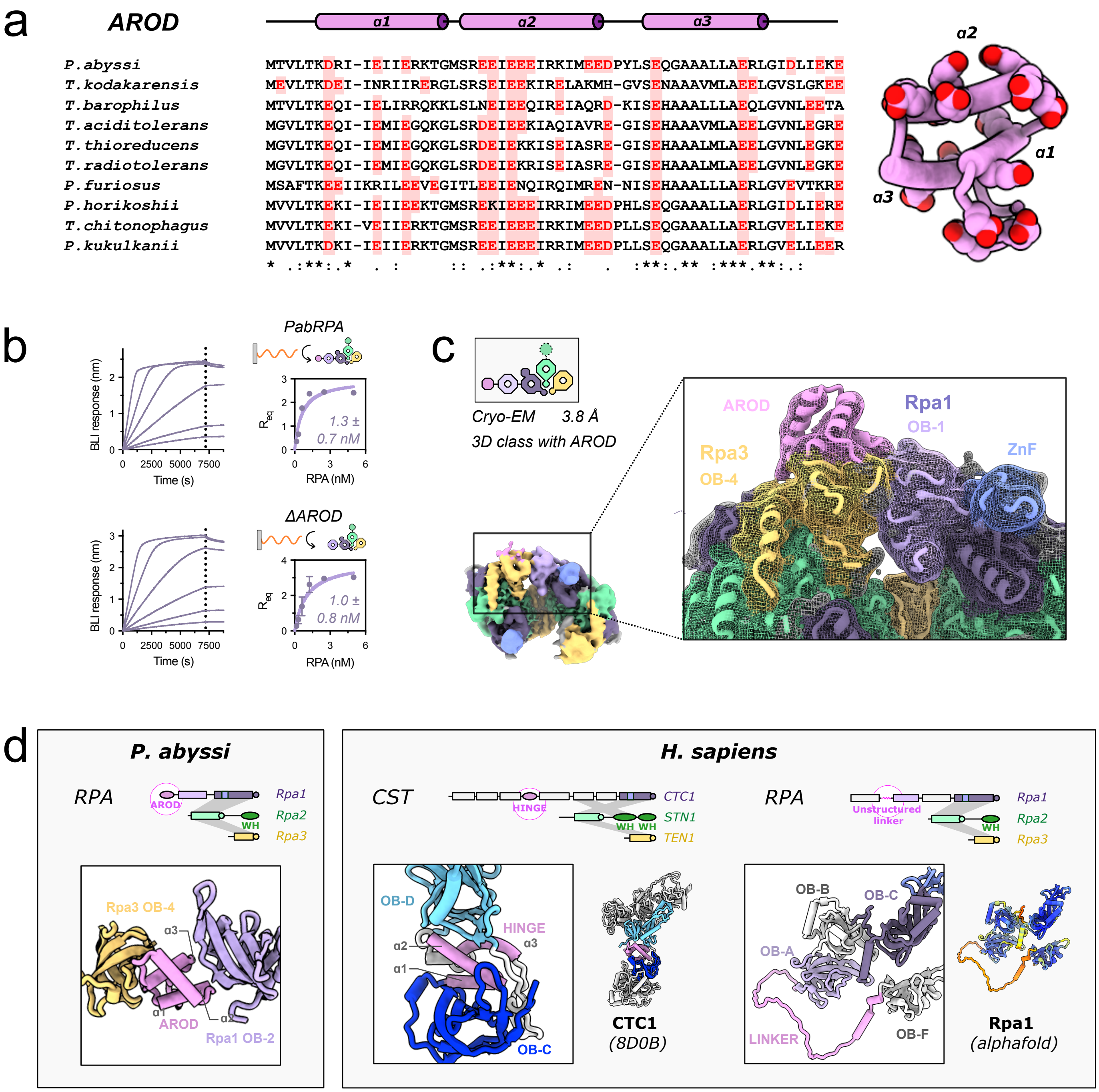

Supplementary figure 13: Sequence, structure and evolution of AROD a. Multiple sequence alignment of AROD from various archaeal species. Aspartates and glutamates are highlighted in red. b. Specific binding of PabRPA and ΔAROD mutant (5, 2.5, 1.25, 0.63, 0.31, 0.16 nM; n=2) to immobilized poly-dT35 ssDNA measured by biolayer interferometry. Steady-state analysis were performed using the average signal measured at the end of the association steps. Data are presented as mean values +/- standard deviations. c. 3.8 Å cryo-EM structure of the tetrameric PabRPA super-structure showing contacts between the Rpa1 N-terminal domains (AROD -OB-1) and Rpa3. d. Comparison of P. abyssi Rpa1 AROD with the three-helices bundle hinge in human CTC1 and the OB-F/OB-A linker in human Rpa1.

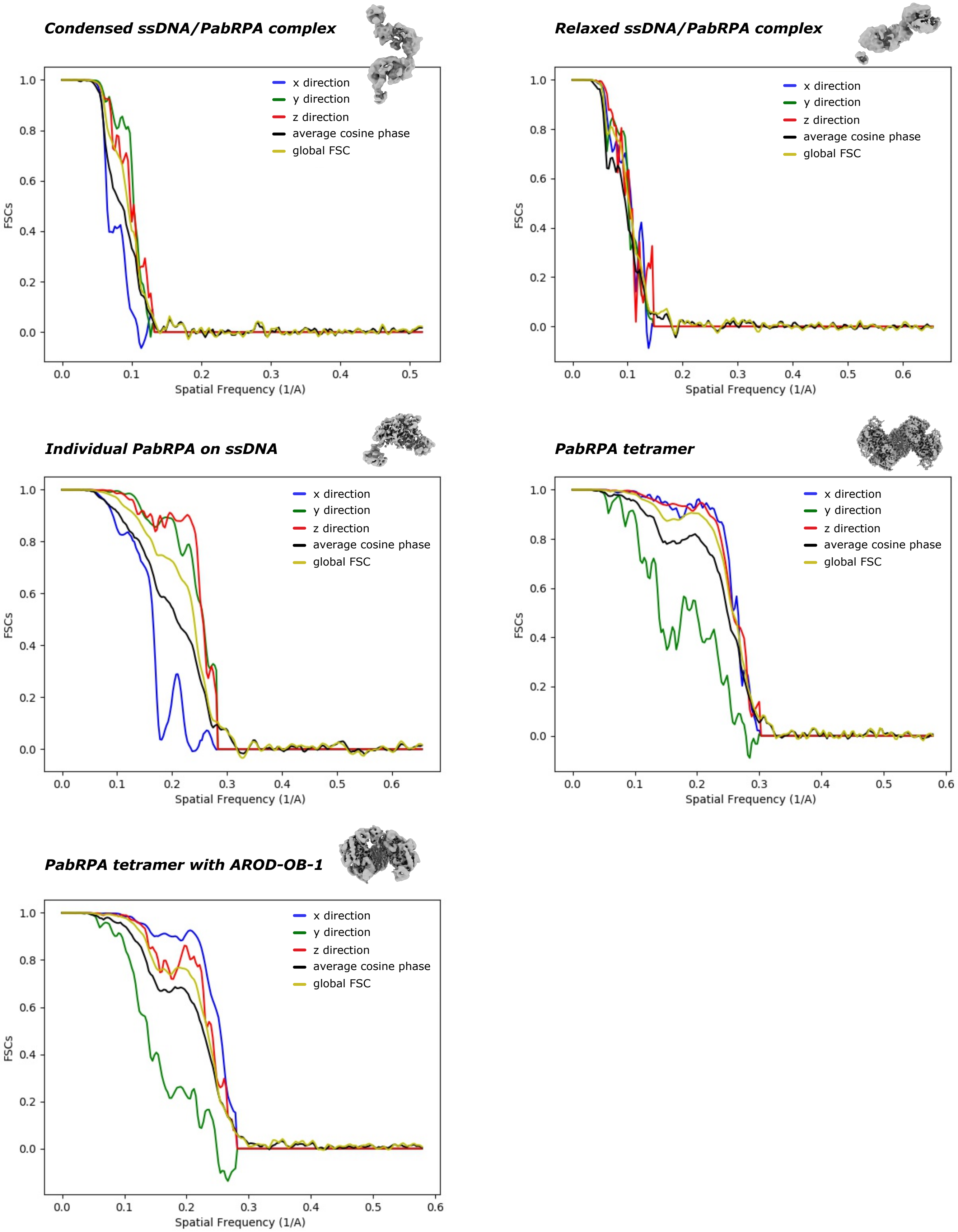

Supplementary figure 14: Directional resolution analysis using 3D Fourier shell correlation. 3D FSC curves were calculated in X, Y and Z directions. Average cosine phase and global FSC curves are also given for each volume.

	**PabRpa1 AROD-OB-1** (8AA9)	**PabRPA** (8AAJ)	**PabRPA Tri-C/d20T** (8AAS)
**Data collection**			
Space group	*P* 2_1	*C* 2 2 2_1	*P* 3_2 2 1
Cell dimensions			
a, *b*, *c* (Å)	52.75, 83.47, 56.19	116.53, 169.74, 201.86	75.23, 75.23, 215.49
α, β, γ (°)	90, 115.3, 90	90, 90, 90	90, 90, 120
Wavelength (Å)	0.97857	0.97857	0.97856
Resolution (Å)	50.0 - 1.8 (1.85 - 1.8)	49.4 - 3.70 (3.80 - 3.70)	48.26 - 3.2 (3.28 - 3.2)
Estimated resolution limit (Å)*	1.76, 1.76, 1.91	5.46, 4.21, 3.26	3.52, 3.52, 3.02
R_{pim}	0.025 (0.986)	0.024 (20.52)	0.016 (2.752)
R_{merge}	0.061 (2.43)	0.123 (89.34)	0.080 (14.31)
$I / \sigma I$	14.6 (0.8)	13.0 (0.1)	22.1 (0.3)
$CC_{1/2}$	0.999 (0.288)	1.000 (0.130)	0.999 (0.223)
Completeness (%)	99.9 (99.5)	99.8 (99.8)	99.9 (99.9)
Redundancy	7.0 (6.9)	28.2 (28.4)	27.1 (27.5)
R_{merge}*	0.054 (1)	0.082 (4.31)	0.067 (4.19)
$I / \sigma I$*	14.6 (1.2)	21.4 (1.0)	27.1 (1.0)
$CC_{1/2}$*	0.999 (0.589)	1.000 (0.542)	1.000 (0.454)
Completeness (%)*	86.9 (50.5)	60.1 (13.3)	80.7 (21.2)
**Refinement**			
Resolution (Å)	32.25 - 1.80 (1.85 - 1.80)	49.4 - 3.70 (3.80 - 3.70)	44.84 - 3.2 (3.28 - 3.2)
No. reflections	37716 (1857)	13191 (1036)	37624 (753)
R_{work} / R_{free} (%)	20.86/22.84	23.41/25.09	26.28/28.90
No. atoms			
Protein	2860	5304	6372
DNA	-	-	
Ligand/ion	2	56	258
Water	290	-	335
B-factors			
Protein	38.6	61.9	51.04
Ligand/ion	76.1	44.13	40.68
Water	50.8	39.49	51.69
R.m.s. deviations			
Bond lengths (Å)	0.008	0.010	0.008
Bond angles (°)	0.95	1.45	1.18

Supplementary table 1: X-ray diffraction data collection and refinement statistics. Values in parentheses are for highest-resolution shell.Dataset from single crystal used per structure. *Values calculated after truncation by STARANISO. Estimated resolution limits along the three crystallographic directions a*, b*, c*.

	**Condensed ssDNA/PabRPA complex** (EMDB-16827) (PDB 8OEL)	**Relaxed ssDNA/PabRPA complex** (EMDB-16826) (PDB 8OEJ)	**Individual PabRPA on ssDNA** (EMDB-16448)	**PabRPA tetramer** (EMDB-16444) (PDB 8C5Y)	**PabRPA tetramer with AROD-OB-1** (EMDB-16445) (PDB 8C5Z)
**Data collection and processing**					
Magnification	150000	150000	150000	150000	150000
Voltage (kV)	200	200	200	300	300
Electron exposure ($e^-/Å^2$)	40	40	40	40	40
Defocus range (μm)	-0.8 to –2.8	-0.8 to –2.8	-0.8 to –2.8	-0.8 to –2.8	-0.8 to –2.8
Pixel size (Å)	0.96	0.76	0.76	0.86	0.86
Symmetry imposed	-	-	-	C2	-
Initial particle images (no.)	847,605	2,403,256	2,403,256	1,761,244	1,761,244
Final particle images (no.)	48,242	34,478	126,856	141,325	41,432
Map resolution (Å)	8.2	8.0	3.9	3.4	3.8
FSC threshold	0.143	0.143	0.143	0.143	0.143
Map resolution range (Å)	7 - 12	7 - 12	3 - 5	3 - 5	3 - 7
**Refinement**					
Initial model used (PDB)	8AA9, 8AAS	8AA9, 8AAS		8AAJ	8AA9, 8AAJ
Model resolution (Å) FSC threshold (0.143)	8.2	8.0		3.3	3.7
Map sharpening *B* factor ($Å^2$)	743	601		130.0	82.4
Model composition					
Non-hydrogen atoms	10024	10024		15198	16616
Protein residues	9462	9462		1868	2044
Nucleotides	560	560			
Ligands	2	2		4	4
Mean B factors ($Å^2$)					
Protein				45.7	278.2
Nucleotides					
Ligand				107.3	291.1
R.m.s. deviations					
Bond lengths (Å)	0.004	0.004		0.004	0.004
Bond angles (°)	0.715	0.702		0.685	0.697
Validation					
MolProbity score	2.10	1.93		1.66	1.75
Clashscore	13.5	12.0		7.98	11.3
Poor rotamers (%)	2.24	1.95		0	0
Ramachandran plot					
Favored (%)	96.79	97.40		96.52	96.92
Allowed (%)	2.99	2.43		3.26	2.8
Disallowed (%)	0.22	0.17		0.22	0.20

Supplementary table 2: Cryo-EM data collection, refinement and validation statistics
