# Supplementary material for: DNA-binding mechanism and evolution of replication protein A
Source: Nat Commun. 2023 Apr 22;14:2326. doi: 10.1038/s41467-023-38048-w (PMC10122647; doi:10.1038/s41467-023-38048-w)
Supplement: Supplementary file 3 — Description of Additional Supplementary Files [file 41467_2023_38048_MOESM3_ESM.pdf]

Description of Additional Supplementary Files

File Name: Supplementary Movie 1
Description: Heterotrimeric structure of the archaeal RPA.

File Name: Supplementary Movie 2
Description: Structure of the RPA tetrameric supercomplex.
